# Interventions facilitating the involvement of relatives of patients with acquired brain injury or malignant brain tumour: A scoping review

**DOI:** 10.1111/jocn.17328

**Published:** 2024-07-30

**Authors:** Mette Gothardt Lundh, Sara Nordentoft, Pernille Sejr Smedegaard, Lena Aadal, Mia Ingerslev Loft, Ingrid Poulsen, Rikke Guldager

**Affiliations:** ^1^ Department of Neurosurgery Copenhagen University Hospital – Rigshospitalet Copenhagen Denmark; ^2^ Hammel Neurorehabilitation and Research Centre Hammel Denmark; ^3^ Department of Clinical Medicine Aarhus University Aarhus Denmark; ^4^ Department of Neurology Copenhagen University Hospital – Rigshospitalet Copenhagen Denmark; ^5^ Research Unit of Nursing and Health Care Aarhus University Aarhus Denmark; ^6^ Department for People and Technology Roskilde University Roskilde Denmark; ^7^ Department of Clinical Research Copenhagen University Hospital – Amager and Hvidovre Hvidovre Denmark

**Keywords:** acquired brain injury, caregivers, intervention, involvement, malignant brain tumour, outcome measurements, outcomes, relatives

## Abstract

**Aim:**

To identify and map the evidence on interventions facilitating the involvement of relatives of patients with an acquired brain injury (ABI) or a malignant brain tumour (MBT).

**Background:**

An ABI or a MBT are severe diseases that have profound impact on the lives of patients and their relatives. The well‐being of the patient may be deteriorated, and relatives may experience a new role and changing caregiving tasks. Involvement of relatives seems essential, and there is a need for identifying interventions facilitating the involvement.

**Design:**

Scoping review.

**Methods:**

The Joanna Briggs Institute methodology was used in this review and the review was reported in accordance with the PRISMA extension for scoping reviews.

**Data Sources:**

The literature search was conducted in MEDLINE, Embase, CINAHL and Cochrane Library. Reference lists of included studies, Google Scholar and Web of Science were also searched.

**Results:**

In total, 46 studies were included of which 36 (78%) involved patients with stroke. Median duration of study interventions were 8 weeks, and nurses were involved as providers of the intervention in 23 (50%) studies. Thirty (65%) studies used a multicomponent intervention. Thirty‐five unique outcomes were identified using 60 unique outcome measurements.

**Conclusion:**

Interventions facilitating the involvement of relatives differed importantly in key characteristics of study interventions, and in relation to the context in which they were used. There was no consensus regarding choice of outcomes and outcome measurements. Our results highlight the complexity of interventions in this field.

**Implications for the Profession and/or Patient Care:**

To our knowledge this is the first scoping review examining interventions facilitating the involvement of relatives of patients with an acquired brain injury or a malignant brain tumour. This review suggests a clear definition of ‘involvement’ in future research and there is a need of development of a core outcome set for use in interventions facilitating the involvement.

**Reporting Method:**

The scoping review was reported in accordance with the PRISMA extension for scoping reviews.

**No Patient or Public Contribution:**

The authors decided to undertake this scoping review without patient and public contribution. However, the protocol was published prior to review conduct and available to the public but we did not receive any comments on it.

**What does this paper contribute to the wider global community?**

- Several interventions for involvement of relatives were identified with a great variety in key characteristics of study interventions.
- Nurses have a central role in performing interventions facilitating involvement of relatives.
- Future studies should clearly define ‘involvement’ and report details of interventions sufficiently.
- There is no consensus regarding outcomes and outcome measurements when assessing interventions facilitating the involvement of relatives of patients and there is a need of developing a core outcome set in this field.

## 1 INTRODUCTION

An acquired brain injury (ABI) or a malignant brain tumour (MBT) are severe diseases that often share similar patient consequences such as physical, psychological, and cognitive disabilities depending on injury or tumour location (Mar et al., 2011; Molassiotis et al., 2010). The well‐being of the patient may be affected including the patient's ability to collaborate with healthcare professionals (HCPs) and participate in decision making about care and treatment throughout the course of disease (Mar et al., 2011). As a result, the patient's relatives may experience a new role and changing caregiving tasks because of the patient becoming more dependent on support and assistance in everyday life (Coco et al., 2011; Piil et al., 2022) and the involvement of relatives seems essential.

Several studies have explored relatives' need for involvement in the care of patients with an ABI or a MBT and a recent scoping review found that the needs of relatives of patients with an ABI were primarily related to information, communication, and support from HCPs (Guldager, Nordentoft, Poulsen, et al., 2023a). Another recent scoping review found that relatives of patients with a MBT identified themselves as already involved but they expressed a need for stronger connection with HCPs because of the rapid disease development and changing caregiving tasks (Guldager, Nordentoft, Poulsen, et al., 2023b). Identifying and meeting relatives' needs for involvement seems essential and may improve patient satisfaction and high‐quality nursing care (Guldager, Nordentoft, Poulsen, et al., 2023a; Guldager, Nordentoft, Poulsen, et al., 2023b). In addition, the HCPs may need this knowledge about relatives' needs for involvement in order to increase the capacity of relatives in their new role as caregiver (Guldager, Nordentoft, Poulsen, et al., 2023a). With the caregiver role the relatives become an active part of the patient's care and their advocacy for the patient plays a significant role since the patient may have difficulties with communication or decision‐making. Facilitating relatives as active members of the treatment team may not only meet relatives' unmet needs (Coco et al., 2011), but may also improve clinical outcomes of the patients (ViBIS, 2015; Sander et al., 2002).

The importance of facilitating the involvement of relatives in care and treatment is widely acknowledged within the healthcare system and has become a political necessity in many countries and healthcare systems around the world (Carman et al., 2013; Harsløf et al., 2019; Vahdat et al., 2014). Nevertheless, little attention has been given to the conceptual meaning of involvement (Thompson, 2007). Involvement, shared decision making, and collaboration are all concepts closely related and frequently used interchangeably (Vahdat et al., 2014). There seems to be no clear definition of involvement which may influence the applicability and carrying out involvement in clinical practice (Owen et al., 2022). There nevertheless seems to be some agreement that involvement implies ‘an active doing’ and a collaboration between patient, relative and HCPs.

Several studies have investigated specific interventions facilitating the involvement of relatives of patients with an ABI or a MBT (Araújo et al., 2018; Avci & Gozum, 2021; Halkett et al., 2023). However, the evidence of interventions facilitating the involvement of relatives of patients with an ABI or a MBT has not previously been mapped.

## 2 AIMS AND OBJECTIVES

This scoping review aimed to identify and map the available evidence on interventions facilitating the involvement of relatives of patients with an ABI or a MBT throughout the course of disease with the following objectives:

1. To describe key characteristics in studies of interventions facilitating the involvement of relatives of patients with an ABI or a MBT.
2. To describe the outcomes and outcome measurements used in studies of interventions facilitating the involvement of relatives of patients with an ABI or a MBT.

## 3 METHODS

### 3.1 Terminology

‘Relative’ was defined as the person providing informal care, which is considered as unpaid care provided to older and/or dependent persons by a person with whom they have a social relationship, for example, spouse, parent or friend (Trintafillou, 2010). ‘Caregiver’ and ‘relative’ are frequently used synonymously in the literature, and studies using the term ‘caregiver’ were also included. The term ‘relative’ was used throughout this review.

### 3.2 Design

This scoping review was conducted according to our published protocol (Guldager, Nordentoft, Aadal, et al., 2023) (see Supplementary Material I—Data S1) which was developed in line with the Joanna Briggs Institute (JBI) methodology for scoping reviews (Peters et al., 2020), and the review is reported in accordance with the Preferred Reporting Items for Systematic reviews and Meta‐Analyses extension for Scoping Reviews (PRISMA‐ScR) Checklist (Tricco et al., 2018) (see Supplementary Material II—Data S1).

### 3.3 Search methods

The following databases were searched: MEDLINE (PubMed), Embase (Ovid), CINAHL (EBSCO) and Cochrane Central Register of Controlled Trials (CENTRAL) in the Cochrane Library (2010 to February 9, 2023). Furthermore, Google Scholar and Web of Science were searched for additional, relevant records (2010 to May 23, 2023). Finally, reference lists of included studies were searched.

The search strategy was developed for PubMed and adapted for the other databases. Relevant index terms were identified, tailored for each database, and used in combination with controlled vocabulary as recommended in the Cochrane Handbook (Lefebvre et al., 2023). The search strategy was developed by the authors in collaboration with an information specialist (see Supplementary Material III—Data S1).

### 3.4 Inclusion and exclusion criteria

Studies including relatives aged 18 years or older of patients aged 18 years or older, with an ABI of any severity or a MBT (WHO Grades 3 or 4 (Louis et al., 2021)) were included. All types of interventions facilitating the involvement of relatives and provided by any HCP (e.g. nurse or physiotherapist) were included. Studies of interventions involving both patients and relatives were included, but studies of interventions involving only patients were excluded. Studies of any outcome were included. Studies conducted in any country and setting (e.g. in‐hospital or community‐based) were included. Only studies in English, German or Scandinavian languages were included for pragmatic reasons. Studies of experimental and observational design, and qualitative and mixed‐methods studies were included. Evidence synthesis such as literature reviews or clinical guidelines were excluded. Protocols of studies that were ongoing or finished but not published, were not included in the review but listed in the ongoing studies' table (see Supplementary Material IV—Data S1).

### 3.5 Study inclusion

The database search results were imported into EndNote 20 (Clarivate Analytics PA, USA) and duplicates were removed in accordance with the methods described by Bramer et al. (2016). Two authors (MGL and RG) independently assessed studies for inclusion in two steps using Covidence (Veritas Health Innovation, Melbourne, Australia). First, titles and abstract were screened for evident exclusions. Second, full‐text records were assessed for final inclusion. Disagreements were resolved through discussion and when consensus could not be reached a third author was consulted (PSS).

### 3.6 Quality appraisal

A formal appraisal of the methodological quality of included studies is generally not performed in scoping reviews (Peters et al., 2020) and was therefore not done.

### 3.7 Data extraction and mapping of results

Two authors (MGL and RG) initially pilot‐tested a data extraction sheet on the first 10 included studies and revised the sheet accordingly. Information was extracted on key study characteristics of study, methods, outcomes, outcome measurements, study interventions and study population (see Supplementary Material V—Data S1). For the placement of intervention in relation to the course of disease, studies were categorised into five phases: (i) Acute treatment; (ii) In‐hospital rehabilitation; (iii) Rehabilitation after discharge; (iv) The continuing developing phase; and (v) Mixed phases (Royal College of Physicians and British Society of Rehabilitation Medicine, 2003). Interventions in the included studies were categorised in three groups regarding target of intervention: (i) Individual; (ii) Group; and (iii) Mixed. When an intervention had an individual target, it was carried out with the HCP and the patient and/or relative. In case the target of the intervention was ‘group’ the intervention was carried out in a group setting with HCP and several patients and/or relatives. The target ‘mixed’ was relevant in case of both individual and group setting in the intervention.

Involvement of relatives were categorised into two groups: (i) Intervention only for relatives; and (ii) Relatives play an active part. This coding refers to the degree of involvement of relatives in the intervention, for example, ‘caregiver‐delivered rehabilitation’ was categorised‘relatives play an active part’. We used the terms ‘outcome’ and ‘outcome measurement’ in line with terminology in the Cochrane Handbook (Li et al., 2023). Study ‘outcome’ refers to an outcome of a particular study (e.g. the primary outcome) and ‘outcome measurement’ the specific instrument or scale used for measuring the effect of the intervention on the specific outcome (e.g. the outcome ‘Caregiver Burden’ was measured using the outcome measurement ‘Caregiver Burden Scale’). Only outcomes and outcome measurements related to relatives were extracted because of the aim of this review. In case a study did not report an outcome but only an outcome measurement, the outcome was coded in accordance with what the outcome measurement originally intended to measure (e.g. the outcome for the outcome measurement ‘Caregiver Strain Index’ would be classified having ‘strain’ as the outcome (Robinson, 1983)).

## 4 RESULTS

### 4.1 Study inclusion

**FIGURE 1:**
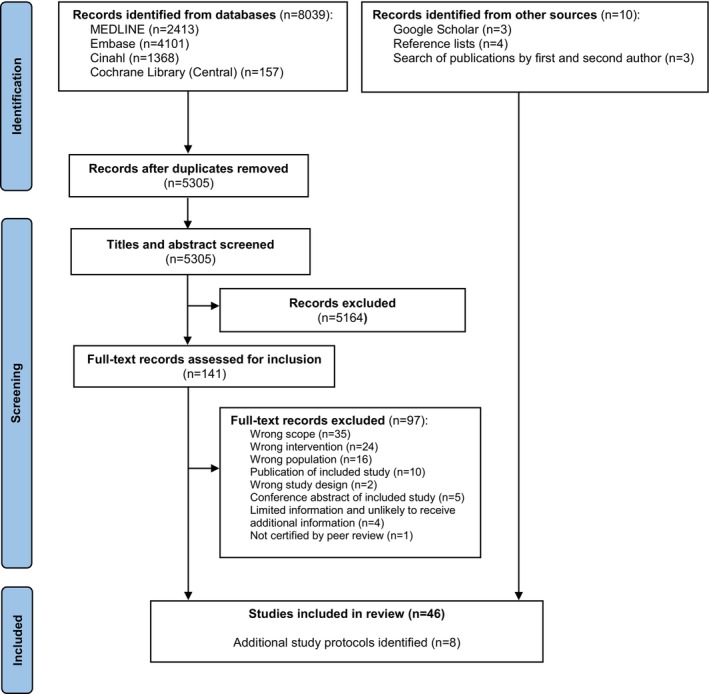
Flow diagram of study inclusion. [Colour figure can be viewed at wileyonlinelibrary.com]

In total 5305 unique records were identified in our database search. By reading titles and abstract we excluded 5164 records and assessed full text of 141 records. Of these records, 97 records were excluded, and 44 records were retained for final assessment. We found additional records for retainment from searching other information sources. Of the 54 retained records 46 studies were included and the remaining eight records were study protocols of ongoing or finished but not published studies (see Figure 1; Supplementary Material IV—Data S1).

### 4.2 Key characteristics of included studies

The median publication year was 2018 (interquartile range [IQR]: 2014–2021) (Table 1; Supplementary Material VI—Data S1). Of the 46 studies, 15 (33%) studies had a corresponding author from Asia, 13 (28%) from Europe and no studies with corresponding author from South America were identified. Only 3 (7%) studies specifically included the term ‘involvement’ in the study aim or objective. The most frequent study designs were randomised controlled trials (RCTs), used in 17 (37%) studies, followed by pilot studies (*n* = 7; 15%). Stroke was the most frequent diagnosis included in 35 (76%) studies. The median number of patients per study was 70 (range: 4–4208) and the median number of relatives per study was 63 (range: 4–4208). Relatives were most often spouses or partners (*n* = 36; 78%) or children (*n* = 25; 54%).

**TABLE 1:** Key characteristics of the 46 included studies.

Study characteristics	Number of studies (%)^a^
Year of publication, median (IQR)	
2018 (2014–2021)	
Continent of corresponding author	
Asia	15 (33)
Europe	13 (28)
North America	10 (22)
Australia	7 (15)
Africa	1 (2)
‘Involvement’ part of aim or objective	3 (7)
Study design	
Randomised controlled trial	17 (37)
Pilot study	7 (15)
Feasibility study	4 (9)
Quasi‐experimental	4 (9)
Qualitative	3 (7)
Non‐randomised controlled trial	2 (4)
Experimental	1 (2)
Mixed methods	1 (2)
Other	7 (15)
Outcome collection methods	
Questionnaire	28 (61)
Interviews and questionnaire	6 (13)
Interviews	3 (7)
Other	9 (20)

Patients' characteristics	Number of studies (%)^a^
Total number, median (range)^b^	70 (4–4208)
Sex (male) %, median (range)^c^	57 (36–86)
Mean age in years (groups)	
30–39	1 (2)
40–49	3 (7)
50–59	4 (9)
60–69	19 (41)
70–79	7 (15)
Not reported or not applicable	12 (26)
Diagnosis	
Stroke	35 (76)
Traumatic brain injury	4 (9)
High‐grade glioma	4 (9)
Acquired brain injury	2 (4)
Stroke and brain injury	1 (2)

Relatives' characteristics	Number of studies (%)^a^
Total number, median (range)^d^	63 (4–4208)
Sex (male) %, median (range)^e^	26 (4–71)
Mean age in years (groups)	
30–39	4 (9)
40–49	5 (11)
50–59	15 (33)
60–69	5 (11)
70–79	1 (2)
Not reported or not applicable	16 (35)
Relation of relative	
Spouse or partner	36^f^ (78)
Child	25 (54)
Parent	11 (24)
Sibling	8 (17)
Other family member	6 (13)
Other relation	22 (48)
Not reported	9 (20)

^a^ Percentages do not always add up to 100 due to rounding.

^b^ Not reported or not applicable in five studies.

^c^ Not reported or not applicable in nine studies.

^d^ Not reported in one study.

^e^ Not reported or not applicable in 13 studies.

^f^ *n* > 37 because ≥1 relation per study.

### 4.3 Key characteristics of study interventions

In 12 (26%) studies the intervention was delivered both in‐hospital and at home. Nine (20%) additional studies delivered interventions solely in‐hospital and 8 (17%) additional studies solely at home (see Table 2; Supplementary Material VII—Data S1). The placement of the intervention in relation to the course of disease varied in included studies with only 1 (2%) study delivering the intervention during the acute treatment, 15 (33%) studies during in‐hospital rehabilitation and 10 (22%) studies during rehabilitation after discharge. The median duration of the intervention was 8 weeks (range: from 1 h up to 52 weeks) and the median length of follow‐up was 4 months (range: 1–12 months). Interventions were performed individually in 23 (50%) studies and mixed (i.e. both individually and in groups) in 8 (17%) studies. In 35 (76%) studies relatives played an active part of the intervention and in 9 (20%) studies the intervention solely involved relatives. None of the included studies measured the level of involvement of relatives. The median number of delivery modes in each study was two (range: 1–3) and they varied between studies with 43 (93%) studies including face‐to‐face delivery and 14 (30%) studies using telephone delivery. Nurses were most often the provider of the intervention, involved in 23 (50%) studies. The median number of components in each study was two (range: 1–6). In 15 (33%) studies, interventions included a single component while in 30 (65%) studies they were multicomponent. Education was present as component in 30 (67%) studies, physical activity, or training in 23 (51%) studies and information in 14 (31%) studies (see Table 3; Supplementary Material VIII—Data S1).

**TABLE 2:** Key characteristics of study interventions (*N* = 46).

Characteristics	Number of studies (%)^a^
Setting	
In‐hospital and home	12 (26)
In‐hospital	9 (20)
Home	8 (17)
Community‐based	2 (4)
Home and community	2 (4)
Outpatient clinic	2 (4)
Other	7 (15)
Not reported	4 (9)
Placement of intervention in relation to course of disease	
Acute treatment	1 (2)
In‐hospital rehabilitation	15 (33)
Rehabilitation after discharge	10 (22)
The continuing developing phase	5 (11)
Mixed phases	11 (24)
Not reported or not applicable	4 (9)
Duration of intervention	
Weeks, median (range)	8 (0^b^‐52)
Variable duration depending on the individual	5 (11)
Not reported	9 (20)
Length of follow‐up	
Months, median (range)	4 (1–12)
None	14 (30)
Not reported	1 (2)
Target of intervention	
Individual	23 (50)
Group	2 (4)
Mixed (individual and group)	8 (17)
Not reported	13 (28)
Involvement of relatives	
Intervention only for relatives	9 (20)
Relatives play an active part	37 (80)
Delivery mode of intervention	
Number in each study, median (range)	2 (1–3)
Person (face‐to‐face)	43 (93)^c^
Telephone	14 (30)
Booklet/written information	10 (22)
Web‐based or online	6 (13)
Email	1 (2)
Video	1 (2)
Not reported	2 (4)
Providers of intervention	
Number in each study, median (range)	1 (1–6)
Nurse	23 (50)^d^
Physiotherapist	11 (24)
Occupational therapist	9 (20)
Neuropsychologist/psychologist	6 (13)
Physician	5 (11)
Social worker	2 (4)
Speech‐language therapist	1 (2)
Other	11 (24)
Not reported	6 (13)
Type of intervention	
Single component (=1 core element)	15 (33)
Multicomponent (≥2 core elements)	30 (65)
Not reported	1 (2)

^a^ Percentages do not always add up to 100 due to rounding.

^b^ The shortest intervention took 1 h.

^c^ *n* > 44 because ≥1 relation per study.

^d^ *n* > 46 because ≥1 mediator per study.

**TABLE 3:** Components in included study interventions.^a^

Component	*n* (%)^b^
Education	30 (67)
Physical activity or training	23 (51)
Information	14 (31)
Emotional or cognitive	9 (20)
Support	9 (20)
Communication	6 (13)
Skills training	4 (9)
Coping	3 (7)
Management or self‐management	3 (7)
Psychological	3 (7)
Goal setting	2 (4)
Social	2 (4)
Cognitive‐behavioural	2 (4)
Coordination of care	2 (4)
Coaching	1 (2)
Problem‐solving	1 (2)
Shared decision making	1 (2)

^a^ One study did not report components in the intervention.

^b^ *n* > 45 because ≥1 component per study.

### 4.4 Relative related outcomes and outcome measurements included in studies

**FIGURE 2:**
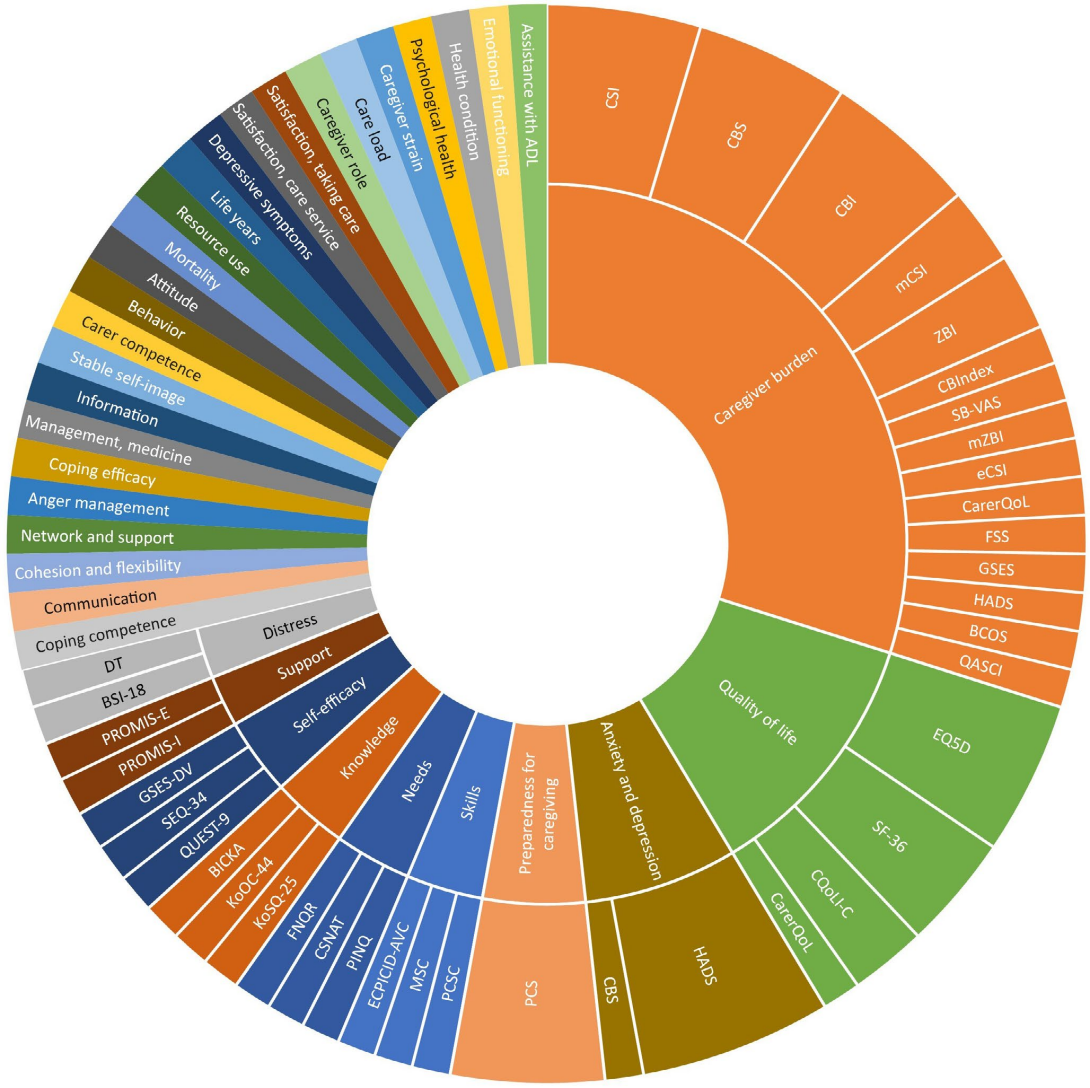
Outcomes and outcome measurements used in studies. The inner circle describes the type of outcome (e.g. Caregiver Burden) and the outer circle the outcome measurement (e.g. Caregiver Burden Scale) used in the included studies. In case of one outcome and one associated outcome measurement, only the outcome is described in the figure (see Supplementary Material X—Data S1 for description of outcome measurement and abbreviation). [Colour figure can be viewed at wileyonlinelibrary.com]

Thirty‐five (76%) studies reported on an outcome related to relatives of which 10 studies reported the outcome as primary outcome, and six studies reported the outcome as secondary outcome. The remaining 19 studies did not report whether outcomes were primary or secondary (see Supplementary Material IX—Data S1). The median number of outcomes per study was two (range: 1–6) and type of outcomes and outcome measurements varied greatly between the included studies. In total 35 unique outcomes were identified with the use of 60 unique outcome measurements. Out of the 35 unique outcomes, 25 only had a single outcome measurement. Caregiver burden was the most common choice of outcome reported in 22 (48%) studies (measured 26 times using 15 unique outcome measurements). Quality of life was reported in 10 (22%) studies (measured 10 times using four unique outcome measurements), anxiety and depression in 5 (11%) studies (measured six times using two unique outcome measurements), and preparedness for caregiving in 4 (9%) studies (measured four times using one unique outcome measurement) (see Figure 2; Supplementary Material X—Data S1).

## 5 DISCUSSION

### 5.1 Summary of findings

Forty‐six studies of interventions facilitating the involvement of relatives of patients with an ABI or a MBT were included in this scoping review. Most interventions involved education, physical activity or training, were delivered by nurses after the acute phase of the disease, and primarily involved relatives to patients with stroke. Although study interventions shared some similarities, they were predominantly heterogenous in relation to the setting, the duration of the interventions, and the components included in the interventions. There seems to be some agreement about the importance of the outcomes ‘caregiver burden’ and ‘quality of life’, and it might be possible that some of the outcomes conceptually overlap, for example, ‘caregiver role’ and ‘carer competence’. Nevertheless, we identified 35 unique outcomes and 60 unique outcome measurements used in the studies. Our findings illustrate the lack of consensus regarding which outcomes are deemed important when evaluating interventions facilitating the involvement of relatives of patients with an ABI or a MBT.

### 5.2 Context

The results from this scoping review are mainly from studies including patients with stroke (i.e. 35 of 46 studies), which may reflect the fact that they make up a significant part of patients with an ABI (Goldman et al., 2022). Further, it shows that interventions are typically tailored for a specific disease rather than the disease group ‘ABI’. Our findings resonate with the findings from a scoping review by de Goumoëns and colleagues from 2018, which included interventions to support families of patients with ABI (de Goumoëns et al., 2018). Although the review included some of the same primary studies as our review, we included more contemporary studies, also assessed interventions for relatives of patients with a MBT and focused on interventions for actively involvement of relatives rather than support.

In the studies included in our review, the level of involvement of relatives varied on a continuum from relatives being included as active participants to relatives being included in an intervention tailored for them as relatives. None of the included studies explicitly described or measured the degree of involvement of relatives. Previous research has assessed and measured family engagement (Goldfarb et al., 2022) but to our knowledge measuring the degree of involvement of relatives is rarely seen in research. The lack of consensus regarding ‘involvement’ might be a reason for the inclusion of the many different outcomes in the studies, which makes comparison between studies challenging. In generally, the study interventions were poorly described which aligns with the results of Hoffmann et al. that reported that essential information about non‐pharmacological interventions are frequently missing in trial publications (Hoffmann et al., 2013).

Overall, there was substantial clinical heterogeneity related to setting, study interventions, and placement of intervention in relation to the course of disease, which reflect that the included study interventions can be defined as complex interventions, since they were mostly multi component interventions with more than one delivery mode (Skivington et al., 2021). Similarly, the included outcomes and outcome measurements, show a high degree of clinical and methodological heterogeneity. However, the study population solely included two specific conditions and this in contrast limits the generalisability of review findings beyond these populations.

### 5.3 Implications for research

It seems essential with involvement of relatives in the treatment and care of patients, especially for patients with an ABI or a MBT due to their possible cognitive impairment (Goldman et al., 2022). However, several of the included studies had cognitive impairment as an exclusion criterion for patients (Araújo et al., 2018; Attard et al., 2018; Chang et al., 2015; Eames et al., 2013; Kim et al., 2013; Lin et al., 2022; Lutz et al., 2022; Nualnetr et al., 2010; Oyesanya et al., 2020; Robinson‐Smith et al., 2016; Taricco et al., 2014; van den Berg et al., 2016; Wang et al., 2015), which seems surprising, since one would assume that they would be most in need of the involvement of relatives. Similarly, some studies also excluded relatives with cognitive impairment, and interventions for facilitating their involvement should likely be designed differently than interventions for relatives without cognitive impairment. There is therefore a need for future interventional studies including patients and relatives with cognitive impairment. Individuals with cognitive impairment are often deemed a vulnerable population and including them in research are encumbered with several ethical and methodological issues (Jones et al., 2021; Kirkevold & Bergland, 2007; Paterson & Scott‐Findlay, 2002). Nevertheless, this subgroup constitutes a significant part of patients with an ABI or a MBT and possibly also their relatives, and they should also be included in future research.

The findings from our review also highlights the poor definition of ‘involvement’ in the studies, and the poor description of study interventions which should be more clearly reported in future studies. In addition, it would be relevant to identify the ideal level of involvement tailored specific to each relative to avoid interventions involving relatives too much or too little.

The identification of several unique outcomes is in accordance with the results from a systematic review in caregiver research in neuro‐oncology that identified 27 different constructs or outcomes (Boele et al., 2022). Our findings highlight the lack of consensus on important outcomes and outcome measurements in interventional research on the involvement of relatives and illustrate a need of developing a core outcome set like what has been developed in other medical areas (Kjaer et al., 2022). Consensus between relatives, HCPs and researchers on which outcomes are important is essential for future clinical studies and evidence synthesis.

Further, while our scoping review methodology does not allow us to make clinical recommendations, we identified 17 RCTs with relevant interventions. A future systematic review with meta‐analysis synthesising results from these trials is needed to guide clinical practice. However, the methodological heterogeneity between relevant trials makes future synthesis of results and thereby the interpretation of results challenging.

### 5.4 Strengths and limitations

To our knowledge this is the first scoping review examining interventions facilitating the involvement of relatives of patients with an ABI or a MBT throughout the disease course. The literature search was comprehensive and carried out in collaboration with an experienced information specialist. Our methods were systematic and besides minor deviations the review was performed in accordance with the published protocol (Peters et al., 2020).

This review also has some limitations. First, despite an extensive literature search it is possible that we might have missed relevant studies. As the aim was to provide a broad overview of the evidence and not to estimate intervention effects, it is unlikely that inclusion of few additional studies would have had a substantial impact on our overall results. The literature search was conducted from 2010 because of the increased focus on involvement of relatives which has changed markedly over the last decade due to among other things, introduction of fast‐track programs and shortened length of hospital stays (Wang et al., 2018). It is possible that relevant studies published prior to 2010 might have been missed. Further, we did not search grey literature comprehensively since we expected that documents such as conference abstracts would not contain information on interventions and outcomes in sufficient level of detail. Second, we did not distinguish between validated and not validated outcome measurements and whether the outcome measurements have good psychometric properties for our study population. We decided prior to review conduct that an actual appraisal was beyond the aim of our review. Given the high degree of heterogeneity in outcome measurements the results of such an appraisal would also have been difficult to interpret. Third, a general challenge was missing data with many studies not reporting essential study characteristics. We judged it to be too extensive to contact study authors for additional information and judged it unlikely that we would be able to retrieve relevant high‐quality data due to general poor reporting in study publications and as most studies were conducted several years ago. Fourth, most of the patients in the studies have stroke, which affects the generalisability of our results to relatives of patients with other diseases such as MBT. However, the findings reflect the current literature in the area and therefore contribute to enlightening the need for further research regarding other patient populations.

## 6 CONCLUSION

In conclusion, a considerable number of studies on interventions facilitating the involvement of relatives of patients with an ABI or a MBT were identified. Most interventions involved education, physical activity, or training, were delivered by nurses after the acute phase of the disease and primarily involved relatives to patients with stroke. The interventions generally differed importantly in relation to the context in which they were used, and which components were included. Although there seems to be some agreement about the importance of the outcomes of caregiver burden and quality of life, there seems to be an overall lack of consensus regarding which outcomes are important. To enable a reliable evaluation of which intervention should be implemented in clinical practice there is a need for developing a core outcome set to be used in future interventional studies.

## FUNDING INFORMATION

This scoping review was supported by the Danish Health Confederation and Danish Regions (Grant number 2657). The funding did not have any influence in the review process.

## CONFLICT OF INTEREST STATEMENT

SN is first author of one of the included studies, she was not involved in study inclusion or data extraction of the study. We declare no additional conflicts of interest.

## Supporting information


Data S1.

## Data Availability

The data that support the findings of this study are available from the corresponding author upon reasonable request.
