# Supplementary material for: Interventions facilitating the involvement of relatives of patients with acquired brain injury or malignant brain tumour: A scoping review
Source: J Clin Nurs. 2024 Jul 30;34(3):784–94. doi: 10.1111/jocn.17328 (PMC11808439; doi:10.1111/jocn.17328)
Supplement: Supplementary file 1 — Data S1. [file JOCN-34-784-s001.docx]

**Supplementary Material I. Deviations from the protocol**

We originally planned using BASE and Grey Matters databases for searching for unpublished studies. However, during our study we decided to use the methods described by Bramer et al. [1] as we found the methods more appropriate. Further, we planned to contact the study authors for missing information but due to the large amount of missing data we decided not to do this. As described in the study protocol we planned involving healthcare professionals and relatives in reviewing the results of the review. However, we found this work to be too methodological comprehensive when included in the review. Instead, we are now planning to conduct a secondary study regarding patient and public involvement of the results of this scoping review.

**Supplementary Material II. Preferred Reporting Items for Systematic reviews and Meta-Analyses extension for Scoping Reviews (PRISMA-ScR) Checklist**

| **SECTION** | **ITEM** | **PRISMA-ScR CHECKLIST ITEM** | **REPORTED ON PAGE #** |
| --- | --- | --- | --- |
| **TITLE** | | | |
| Title | 1 | Identify the report as a scoping review. | Title page |
| **ABSTRACT** | | | |
| Structured summary | 2 | Provide a structured summary that includes (as applicable): background, objectives, eligibility criteria, sources of evidence, charting methods, results, and conclusions that relate to the review questions and objectives. | Abstract page |
| **INTRODUCTION** | | | |
| Rationale | 3 | Describe the rationale for the review in the context of what is already known. Explain why the review questions/objectives lend themselves to a scoping review approach. | Introduction page |
| Objectives | 4 | Provide an explicit statement of the questions and objectives being addressed with reference to their key elements (e.g., population or participants, concepts, and context) or other relevant key elements used to conceptualize the review questions and/or objectives. | Introduction page |
| **METHODS** | | | |
| Protocol and registration | 5 | Indicate whether a review protocol exists; state if and where it can be accessed (e.g., a Web address); and if available, provide registration information, including the registration number. | Methods, section 3.2 Supplementary Material I |
| Eligibility criteria | 6 | Specify characteristics of the sources of evidence used as eligibility criteria (e.g., years considered, language, and publication status), and provide a rationale. | Methods, section 3.4 |
| Information sources | 7 | Describe all information sources in the search (e.g., databases with dates of coverage and contact with authors to identify additional sources), as well as the date the most recent search was executed. | Methods, section 3.3 |
| Search | 8 | Present the full electronic search strategy for at least 1 database, including any limits used, such that it could be repeated. | Methods, section 3.4 Supplementary Material III |
| Selection of sources of evidence | 9 | State the process for selecting sources of evidence (i.e., screening and eligibility) included in the scoping review. | Methods, section 3.5 |
| Data charting process | 10 | Describe the methods of charting data from the included sources of evidence (e.g., calibrated forms or forms that have been tested by the team before their use, and whether data charting was done independently or in duplicate) and any processes for obtaining and confirming data from investigators. | Methods, section 3.7 Supplementary Material V |
| Data items | 11 | List and define all variables for which data were sought and any assumptions and simplifications made. | Methods, section 3.4 |
| Critical appraisal of individual sources of evidence | 12 | If done, provide a rationale for conducting a critical appraisal of included sources of evidence; describe the methods used and how this information was used in any data synthesis (if appropriate). | Methods, section 3.6 |
| Synthesis of results | 13 | Describe the methods of handling and summarizing the data that were charted. | Methods, section 3.7 |
| **RESULTS** | | | |
| Selection of sources of evidence | 14 | Give numbers of sources of evidence screened, assessed for eligibility, and included in the review, with reasons for exclusions at each stage, ideally using a flow diagram. | Results, section 4.1  Figure 1 |
| Characteristics of sources of evidence | 15 | For each source of evidence, present characteristics for which data were charted and provide the citations. | Supplementary Material VI, VII, VIII, IX, X |
| Critical appraisal within sources of evidence | 16 | If done, present data on critical appraisal of included sources of evidence (see item 12). | Methods, section 3.6 |
| Results of individual sources of evidence | 17 | For each included source of evidence, present the relevant data that were charted that relate to the review questions and objectives. | Results, section 4.2, 4.3 and 4.4 Table 1, 2 and 3 Figure 2 |
| Synthesis of results | 18 | Summarize and/or present the charting results as they relate to the review questions and objectives. | Results, section 4.2, 4.3 and 4.4 Table 1, 2 and 3 Figure 2 |
| **DISCUSSION** | | | |
| Summary of evidence | 19 | Summarize the main results (including an overview of concepts, themes, and types of evidence available), link to the review questions and objectives, and consider the relevance to key groups. | Discussion, section 5, 5.1, 5.2 and 5.3 |
| Limitations | 20 | Discuss the limitations of the scoping review process. | Discussion, section 5.4 |
| Conclusions | 21 | Provide a general interpretation of the results with respect to the review questions and objectives, as well as potential implications and/or next steps. | Conclusion, section 6 |
| **FUNDING** | | | |
| Funding | 22 | Describe sources of funding for the included sources of evidence, as well as sources of funding for the scoping review. Describe the role of the funders of the scoping review. | Title page |

**Supplementary Material III. Search strategy**

**MEDLINE (PubMed)**

| **#** | **Search** |
| --- | --- |
| 1 | acquired brain injur*[tw] |
| 2 | brain injur*[tw] |
| 3 | brain injuries[mesh] |
| 4 | stroke[tw] |
| 5 | stroke[mesh] |
| 6 | glioblastoma*[tw] |
| 7 | brain cancer*[tw] |
| 8 | brain neoplasms[tw] |
| 9 | brain neoplasms[mesh] |
| 10 | malignant brain tumor*[tw] |
| 11 | malignant brain tumour*[tw] |
| 12 | glioma*[tw] |
| 13 | glioma[mesh] |
| 14 | #1 OR #2 OR #3 OR #4 OR #5 OR #6 OR #7 OR #8 OR #9 OR #10 OR #11 OR #12 OR #13 |
| 15 | intervention[tw] |
| 16 | measure*[tw] |
| 17 | measurement*[tw] |
| 18 | scale*[tw] |
| 19 | instrument*[tw] |
| 20 | assessment*[tw] |
| 21 | knowledge*[tw] |
| 22 | knowledge[mesh] |
| 23 | education[tw] |
| 24 | education[mesh] |
| 25 | information[tw] |
| 26 | #15 OR #16 OR #17 OR #18 OR #19 OR #20 OR #21 OR #22 OR #23 OR #24 OR #25 |
| 27 | relative*[tw] |
| 28 | famil*[tw] |
| 29 | family[mesh] |
| 30 | caregiver*[tw] |
| 31 | caregiver[mesh] |
| 32 | carer*[tw] |
| 33 | family relation*[tw] |
| 34 | family relations[mesh] |
| 35 | spouse*[tw] |
| 36 | spouses[mesh] |
| 37 | wife*[tw] |
| 38 | husband*[tw] |
| 39 | partner*[tw] |
| 40 | sibling*[tw] |
| 41 | siblings[mesh] |
| 42 | brother*[tw] |
| 43 | sister*[tw] |
| 44 | kinship*[tw] |
| 45 | next of kin*[tw] |
| 46 | #27 OR #28 OR #29 OR #30 OR #31 OR #32 OR #33 OR #34 OR #35 OR #36 OR #37 OR #38 OR #39 OR #40 OR #41 OR #42 OR #43 OR #44 OR #45 |
| 47 | involvement[tw] |
| 48 | needs[tw] |
| 49 | decision making[mesh] |
| 50 | decision making[tw] |
| 51 | decision making, shared[mesh] |
| 52 | continuity of patient care[mesh] |
| 53 | continuity of patient care[tw] |
| 54 | patient participation[mesh] |
| 55 | patient participation[tw] |
| 56 | family practice[mesh] |
| 57 | family practice[tw] |
| 58 | collaboration*[tw] |
| 59 | #47 OR #48 OR #49 OR #50 OR #51 OR #52 OR #53 OR #54 OR #55 OR #56 OR #57 OR #58 |
| 60 | #14 AND #26 AND #46 AND #59 |
| 61 | #60 with filters applied: Year 2010-current, Danish, English, German, Norwegian, Swedish, Adult: 19+ years |

**Embase (Ovid)**

| **#** | **Search** |
| --- | --- |
| 1 | ”acquired brain injur*”.tw. |
| 2 | ”brain injur*”.tw. |
| 3 | exp brain injury/ |
| 4 | stroke.tw. |
| 5 | exp cerebrovascular accident/ |
| 6 | glioblastoma*.tw. |
| 7 | exp glioblastoma/ |
| 8 | ”brain cancer*”.tw. |
| 9 | brain neoplasms.tw. |
| 10 | exp brain tumor/ |
| 11 | ”malignant brain tumor*”.tw. |
| 12 | ”malignant brain tumour*”.tw. |
| 13 | ”glioma*”.tw. |
| 14 | exp glioma/ |
| 15 | 1 or 2 or 3 or 4 or 5 or 6 or 7 or 8 or 9 or 10 or 11 or 12 or 13 or 14 |
| 16 | intervention.tw. |
| 17 | exp intervention study/ |
| 18 | exp nursing intervention/ |
| 19 | ”measure*”.tw. |
| 20 | ”measurement*”.tw. |
| 21 | ”scale*”.tw. |
| 22 | ”instrument*”.tw. |
| 23 | ”assessment*”.tw. |
| 24 | ”knowledge*”.tw. |
| 25 | exp knowledge/ |
| 26 | education.tw. |
| 27 | exp education/ |
| 28 | information.tw. |
| 29 | 16 or 17 or 18 or 19 or 20 or 21 or 22 or 23 or 24 or 25 or 26 or 27 or 28 |
| 30 | “relative*”.tw. |
| 31 | exp relative/ |
| 32 | “famil*”.tw. |
| 33 | exp family/ |
| 34 | “caregiver*”.tw. |
| 35 | exp caregiver/ |
| 36 | “carer*”.tw. |
| 37 | “family relation*”.tw. |
| 38 | exp family relation/ |
| 39 | “spouse*”.tw. |
| 40 | exp spouse/ |
| 41 | “wife*”.tw. |
| 42 | “husband*”.tw. |
| 43 | “partner*”.tw. |
| 44 | “sibling*”.tw. |
| 45 | exp sibling/ |
| 46 | “brother*”.tw. |
| 47 | “sister*”.tw. |
| 48 | “kinship*”.tw. |
| 49 | “next of kin*”.tw. |
| 50 | 30 or 31 or 32 or 33 or 34 or 35 or 36 or 37 or 38 or 39 or 40 or 41 or 42 or 43 or 44 or 45 or 46 or 47 or 48 or 49 |
| 51 | involvement.tw. |
| 52 | needs.tw. |
| 53 | exp decision making/ |
| 54 | decision making.tw. |
| 55 | exp patient care/ |
| 56 | continuity of patient care.tw. |
| 57 | exp patient participation/ |
| 58 | patient participation.tw. |
| 59 | family practice.tw. |
| 60 | collaboration*.tw. |
| 61 | 51 or 52 or 53 or 54 or 55 or 56 or 57 or 58 or 59 or 60 |
| 62 | 15 and 29 and 50 and 61 |
| 63 | limit 62 to ((danish or english or german or norwegian or swedish) and yr=”2010 -Current” and (adult <18 to 64 years> or aged <65+ years)) |

**CINAHL (EBSCO)**

| **#** | **Search** |
| --- | --- |
| S1 | acquired brain injur* |
| S2 | brain injur* |
| S3 | (MH ”Brain Injuries+”) |
| S4 | stroke |
| S5 | (MH ”Stroke+”) |
| S6 | glioblastoma* |
| S7 | brain cancer* |
| S8 | brain neoplasms |
| S9 | (MH ”Brain neoplasms+”) |
| S10 | malignant brain tumor* |
| S11 | malignant brain tumour* |
| S12 | glioma* |
| S13 | (MH ”Glioma+”) |
| S14 | #S1 or #S2 or #S3 or #S4 or #S5 or #S6 or #S7 or #S8 or #S9 or #S10 or #S11 or #S12 or #S13 |
| S15 | intervention |
| S16 | (MH ”Internet-based Intervention”) OR (MH “Psychosocial Intervention”) OR (MH “Nursing Interventions”) |
| S17 | measure* |
| S18 | measurement* |
| S19 | scale* |
| S20 | instrument* |
| S21 | assessment* |
| S22 | knowledge* |
| S23 | (MH ”Knowledge+”) |
| S24 | education |
| S25 | (MH ”Education+”) |
| S26 | information |
| S27 | S15 or S16 or S17 or S18 or S19 or S20 or S21 or S22 or S23 or S24 or S25 or S26 |
| S28 | relative* |
| S29 | famil* |
| S30 | (MH “Family+”) |
| S31 | caregiver* |
| S32 | (MH "Caregiver Burden") OR (MH "Caregiver Attitudes") OR (MH "Caregiver Support") OR (MH "Caregiver-Patient Relationship (Iowa NOC)") OR (MH "Caregiver Well-Being (Iowa NOC)") OR (MH "Caregiver Support (Iowa NIC)") OR (MH "Caregiver Physical Health (Iowa NOC)") OR (MH "Caregiver Emotional Health (Iowa NOC)") |
| S33 | carer* |
| S34 | family relation* |
| S35 | (MH “Family Relations+”) |
| S36 | spouse* |
| S37 | (MH “Spouses”) |
| S38 | wife* |
| S39 | husband* |
| S40 | partner* |
| S41 | sibling* |
| S42 | (MH “Siblings”) |
| S43 | brother* |
| S44 | sister* |
| S45 | kinship* |
| S46 | next of kin |
| S47 | S28 or S29 or S30 or S31 or S32 or S33 or S34 or S35 or S36 or S37 or S38 or S39 or S40 or S41 or S42 or S43 or S44 or S45 or S46 |
| S48 | involvement |
| S49 | needs |
| S50 | (MH “Decision Making”+) |
| S51 | decision making |
| S52 | (MH “Continuity of Patient Care+”) |
| S53 | continuity of patient care |
| S54 | patient participation |
| S55 | family practice |
| S56 | (MH “Family Practice”) |
| S57 | collaboration* |
| S58 | S48 or S49 or S50 or S51 or S52 or S53 or S54 or S55 or S56 or S57 |
| S59 | S14 and S27 and S47 and S58 |
| S60 | S59 with limiters: Published date 20100101-current, danish, german, english, all adult |

**Cochrane Central Register of Controlled Trials (CENTRAL) in the Cochrane Library**

| **#** | **Search** |
| --- | --- |
| 1 | (acquired brain injur*):kw |
| 2 | (brain injur*):kw |
| 3 | MeSH descriptor: [Brain Injuries] explode all trees |
| 4 | (stroke):kw |
| 5 | MeSH descriptor: [Stroke] explode all trees |
| 6 | (glioblastoma*):kw |
| 7 | (brain cancer*):kw |
| 8 | (brain neoplasms):kw |
| 9 | (malignant brain tumor*):kw |
| 10 | (malignant brain tumour*):kw |
| 11 | (glioma*):kw |
| 12 | MeSH descriptor: [Glioma] explode all trees |
| 13 | #1 OR #2 OR #3 OR #4 OR #5 OR #6 OR #7 OR #8 OR #9 OR #10 OR #11 OR #12 |
| 14 | (intervention):kw |
| 15 | (measure*):kw |
| 16 | (measurement*):kw |
| 17 | (scale*):kw |
| 18 | (instrument*):kw |
| 19 | (assessment*):kw |
| 20 | (knowledge*):kw |
| 21 | MeSH descriptor: [Knowledge] explode all trees |
| 22 | (education):kw |
| 23 | MeSH descriptor: [Education] explode all trees |
| 24 | (information):kw |
| 25 | #14 OR #15 OR #16 OR #17 OR #18 OR #19 OR #20 OR #21 OR #22 OR #23 OR #24 |
| 26 | (relative*):kw |
| 27 | (famil*):kw |
| 28 | MeSH descriptor: [Family] explode all trees |
| 29 | (caregiver*):kw |
| 30 | MeSH descriptor: [Caregivers] explode all trees |
| 31 | (carer*):kw |
| 32 | (family relation*):kw |
| 33 | (spouse*):kw |
| 34 | (wife*):kw |
| 35 | (husband*):kw |
| 36 | (partner*):kw |
| 37 | (sibling*):kw |
| 38 | (brother*):kw |
| 39 | (sister*):kw |
| 40 | (kinship*):kw |
| 41 | #26 OR #27 OR #28 OR #29 OR #30 OR #31 OR #32 OR #33 OR #34 OR #35 OR #36 OR #37 OR #38 OR #39 OR #40 |
| 42 | (involvement):kw |
| 43 | (needs):kw |
| 44 | MeSH descriptor: [Decision Making] explode all trees |
| 45 | (decision making):kw |
| 46 | MeSH descriptor [Continuity of Patient Care] explode alle trees |
| 47 | (continuity of patient care):kw |
| 48 | MeSH descriptor: [Patient Participation] explode all trees |
| 49 | (patient participation):kw |
| 50 | MeSH descriptor: [Family Practice] explode all trees |
| 51 | (family practice):kw |
| 52 | (collaboration*):kw |
| 53 | #42 OR #43 OR #44 OR #45 OR #46 OR #47 OR #48 OR #49 OR #50 OR #51 OR #52 |
| 54 | #13 AND #25 AND #41 AND #53 |
| 55 | #54 with applied search limits: Publication Year from 2010 to present |

**Google Scholar**

| **#** | **Search**^a^ **(Find articles)** |
| --- | --- |
| 1 | involvement caregiver [with all of the words]  brain injury [with the exact phrase] |
| 2 | Intervention caregiver [with all of the words]  brain injury [with the exact phrase] |
| 3 | involvement caregiver stroke [with all of the words] |
| 4 | involvement caregiver [with all of the words]  brain cancer [with the exact phrase] |
| 5 | involvement caregiver [with all of the words]  brain tumor [with the exact phrase] |
| 6 | involvement caregiver [with all of the words] |

^a^ Limitations for the search: 1. “where my words occur”: anywhere in the article and 2. “Return articles dated between”: 2010-present

**Web of Science**

| **#** | **Search**^a^ **(Web of Science Core Collection)** |
| --- | --- |
| 1 | brain injur* [Topic] |
| 2 | stroke [Topic] |
| 3 | glioblastoma* [Topic] |
| 4 | brain cancer [Topic] |
| 5 | brain tum$r [Topic] |
| 6 | glioma [Topic] |
| 7 | relative* [Topic] |
| 8 | family [Topic] |
| 9 | caregiver* [Topic] |
| 10 | spouse* [Topic] |
| 11 | involvement [Topic] |
| 12 | needs [Topic] |
| 13 | intervention* [Topic] |

^a^ Index Date: 2010-01-01 to 2023-05-23

**Supplementary Material IV. Ongoing studies’ table (n=8)**

| **KEY CHARACTERISTICS OF STUDY** | | | | | | | | | | | | **KEY CHARACTERISTICS OF INTERVENTION** | | | | | | | | | | |
| --- | --- | --- | --- | --- | --- | --- | --- | --- | --- | --- | --- | --- | --- | --- | --- | --- | --- | --- | --- | --- | --- | --- |
| Author and year of publication | Country of corresponding author | | | Continent of corresponding author | Aim/objective | | | Study design &  outcome collection method | | Diagnosis of patients | | Setting | | Duration of intervention | | Target of intervention | | Delivery mode of intervention | | Providers of intervention | | Type of intervention |
| Blanton 2019 [2] | USA | | | North America | The primary objective of this trial is to evaluate feasibility of a web-based, family-focused intervention for stroke survivors and their care partners | | | Study protocol Interviews and questionnaire | | Stroke | | Community-based | | 4-6 weeks | | Individual | | Person (face-to-face) Telephone Web-based/online | | PT^a^ OT^b^ | | Multi-compontent |
| Brasier 2016 [3] | Australia | | | Australia | The aim of the study is to determine whether a stroke-specific Optimal Health Program improves the psychosocial health of stroke survivors and their carers, compared to usual care | | | Study protocol Questionnaire | | Stroke | | A nominated place of convenience by the participant | | 5 months | | Mixed | | Person (face-to-face) | | Nurse NP or P^c^ | | Single compontent |
| Elsheikh 2020 [4] | Egypt | | | Asia | The proposed study aims to evaluate the effect of a tailored multidimensional intervention on the care burden among family caregivers of stroke survivors | | | Study protocol Questionnaire | | Stroke | | Home | | 6 months | | Mixed | | Person (face-to-face) | | Nurse | | Multi-compontent |
| Mulder 2022 [5] | The Netherlands | | | Europe | The Armed4Stroke study aims to investigate the effects of a caregiver-mediated exercise program using a blended care approach in addition to usual care, on recovery of mobility in the first 6 months poststroke | | | Study protocol Questionnaire | | Stroke | | Home | | 8 weeks | | Individual | | Web-based/online | | PT | | Single compontent |
| Ownsworth 2019 [6] | Australia | | | Australia | This paper describes the protocol for a pragmatic randomised control trial (RCT) evaluating the clinical efficacy and cost‐effectiveness of the Making Sense of Brain Tumour program delivered via telehealth (Tele‐MAST) relative to standard care | | | Study protocol Questionnaire | | Primary brain tumor | | Home | | 10 weeks | | Individual | | Web-based/online | | NP or P | | Multi-compontent |
| Vloothuis 2015 [7] | The Netherlands | | | Europe | The primary aim of the CARE4STROKE study is to evaluate the effects and cost-effectiveness of a caregiver-mediated exercises program combined with e-health services after stroke in terms of self-reported mobility and length of stay | | | Study protocol Interviews and questionnaire | | Stroke | | In-hospital, stroke unit, rehabilitation center, or nursing home | | 8 weeks | | Individual | | Person (face-to-face) | | PT | | Single compontent |
| Worrall 2016 [8] | | Australia | Australia | | | The study aim is to determine whether an early intervention, Aphasia ASK, for the person with aphasia after stroke and their family members leads to better mood and quality of life outcomes for people with aphasia, less caregiver burden, and better mental health for family members compared to an attention-control intervention (Secondary Stroke Prevention Information Program; SSPIP) at 12 months post stroke | Study protocol Questionnaire | | Stroke | | Home and community | | 6 weeks | | Individual | | Person (face-to-face) | | SLT^d^ | | Multi-compontent | |
| Yan  2016 [9] | | China | Asia | | | This study aims to develop and implement a simplified stroke rehabilitation program that utilizes nurses and family caregivers for service delivery and evaluate its feasibility and effectiveness in rural China | Study protocol Focus groups, observations, and interviews | | Stroke | | In-hospital and home | | Individually, depending on duration of admission | | Individual | | Person (face-to-face) | | Nurse | | Multi-compontent | |

^a^ PT: Physiotherapist
^b^ OT: Occupational therapist
^c^ NP or P: Neuropsychologist or psychologist
^d^ SLT: Speech-language therapist

**Supplementary Material V. Data extraction tool**

We extracted data in Microsoft Excel 2016 (Redmond, Washington, USA). Because of the transparency we list the variables extracted instead of reporting them in tabular.

**STUDY ID**

**KEY CHARACTERISTICS OF STUDY**

- Year of publication
- Journal
- Country of origin
- Continent of corresponding author
- Aim/objective
- Is ‘involvement’ present (verbatim) in the aim/objective?

**METHODS**

- Study design
- Outcome collection methods

**OUTCOMES AND OUTCOME MEASUREMENTS**

- Order of outcome
- Outcome measurements

**KEY CHARACTERISTICS OF STUDY INTERVENTIONS**

- Setting
- Type of intervention
- Placement of intervention in relation to course of disease
- Number of components in intervention
- Components
- Duration of intervention
- Length of follow-up
- Intervention design
- Delivery mode of intervention
- Providers of intervention
- Provided by
- Description of intervention (verbatim)

**STUDY POPULATION**

- Total number of patients
- Total number of caregivers
- Patient age (mean)
- Patient age (indexed in groups)
- Patient diagnosis
- Patient gender (male, %)
- Caregiver age (mean)
- Caregiver age (indexed in groups)
- Caregiver gender (male, %)
- Caregiver relation

**Supplementary Material VI. Key characteristics of included studies (n=46)**

| **KEY CHARACTERISTICS OF STUDY** | | | | | **STUDY POPULATION** | | | | | | |  |
| --- | --- | --- | --- | --- | --- | --- | --- | --- | --- | --- | --- | --- |
|  |  |  |  |  |  | Total number of participants | | Age in mean | | Gender, male (%) | | Relation of relative |
| Author and year of publication | Country of corre-sponding author | Continent of corre-sponding origin | Aim/objective | Study design &  outcome collection method | Diagnosis of patients | Pa-  tients | Rela-tives | Pa-  tients | Rela-tives | Pa-  tients | Rela-  tives |  |
| Aguirrezabal 2013 [10] | Spain | Europe | To evaulate the effect of a post-stroke information and carer training intervention provided in the rehabilitation hospital setting on patient and carers' satisfaction | Non-randomised controlled trial Questionnaire | Stroke | 150 | 158 | 66.7 | 59.8 | 70 | 20.3 | Child  Spouse or partner  Other relation |
| Araújo 2018 [11] | Portugal | Europe | Describing an intervention of informal caregivers' skills training when taking care of older people after a stroke | Quasi-experimental  Questionnaire | Stroke | NA^a^ | 174 | NA | 55.2 | NA | 10.9 | Child  Spouse or partner  Other family member  Other relation |
| Attard 2018 [12] | Australia | Australia | To determine potential efficacy of a speech-language pathologist- and social worker-led community aphasia group-model for four people with aphasia and their spouses on living well with aphasia within a proof-of-concept trial | Pilot study  Interviews and questionnaire | Stroke | 4 | 4 | 63.8 | 60.3 | 75 | 25 | Spouse or partner |
| Avci 2021 [13] | Turkey | Europe | To explore caregivers’ experience with the TEMpEST discharge program | Qualitative Interviews | Stroke | NA | 23 | NA | 33 | NA | 13 | Parent Spouse or partner  Other family member |
| Berg 2016 [14] | Australia | Australia | This proof-of-concept trial investigated the effects of an 8-week program of caregiver-mediated exercises commenced in hospital combined with tele-rehabilitation services on patient self-reported mobility and caregiver burden | Pilot study Other (questionnaire, measurements of activity) | Stroke | 63 | 63 | 68.7 | NA | 63.5 | NA | Child  Parent  Spouse or partner |
| Chang 2015 [15] | South Korea | Asia | Examine the effects of a family involvement and individually tailored functional rehabilitation program in adult day care centers on elderly Korean stroke patients' perceived health, activities of daily living, instrumental activities of daily living, and cost of health services, and on their family caregivers' satisfaction | Other (pre- and posttest design) Questionnaire | Stroke | 19 | 19 | 74.5 | 61.5 | 52.6 | 21.1 | Child  Spouse or partner  Other family member |
| Chu 2020 [16] | China | Asia | To implement and evaluate an innovative rehabilitation program that utilizes nurses and family member caregivers for the service delivery for disabled stroke patients in rural regions of Chongqing, Southwest China | RCT Questionnaire | Stroke | NR^b^ | NR | NR | NR | NR | NR | NR |
| Daviet 2022 [17] | France | Europe | The main objective of this work was to examine whether there is an improvement in the social participation of post-stroke patients (on the subacute and chronic phase) who received a rehabilitation program provided by CSRT. The secondary objectives were also to show an improvement in the patients’ quality of life and a reduction in the caregiver burden | Other (cohort study, pragmatic without control group) Questionnaire | Stroke | 70 | 136 | NR | NR | NR | NR | NR |
| Deyhoul 2019 [18] | Iran | Asia | The study evaluated the effect of the family-centered empowerment program on the ability of Iranian patients with stroke to perform activities of daily 76living, as well as on family caregiver burden | RCT Questionnaire | Stroke | 90 | 90 | 67 | 40.9 | 58.9 | 3.6 | Child  Sibling  Spouse or partner |
| Eames 2013 [19] | Australia | Australia | This study aimed to evaluate the effects of an education package which utilised both strategies on the kn40owledge, health and psychosocial outcomes of stroke patients and carers | RCT Questionnaire | Stroke | 76 | 61 | NR | NR | NR | NR | Child  Sibling  Spouse or partner  Other relation |
| Einerson 2022 [20] | USA | North America | This study assesses the feasibility and acceptability of GETCare, a remote Goal-based Education and skills Training program | Pilot study Interviews and questionnaire | Stroke | 28 | 28 | 57.6 | 51.1 | 78.6 | 17.9 | Child  Spouse or partner  Other relation |
| Forster 2013 [21] | United Kingdom | Europe | The TRACS trial investigated a training programme for caregivers (the London Stroke Carers Training Course, LSCTC) on physical and psychological outcomes, including cost-eff effectiveness, for patients and caregivers after a disabling stroke | RCT Questionnaire | Stroke | 928 | 928 | 71.2 | 60.9 | 55.9 | 31.5 | Child  Spouse or partner  Other relation |
| Galvin 2014 [22] | Ireland | Europe | To explore the impact of family involvement in exercise delivery after stroke from the perspective of the individual with stroke and his or her family member | Qualitative Interviews | Stroke | 40 | 15 | 66.6 | NR | 50 | NR | Child  Spouse or partner |
| Halkett 2023 [23] | Australia | Australia | To deliver a nurse-led intervention (Care-IS) to carers to improve their preparedness to care and reduce distress | RCT Questionnaire | High-grade glioma | 188 | 188 | 60.1 | 57.1 | 71.8 | 25.5 | Child  Parent  Spouse or partner  Other relation |
| Harris 2010 [24] | Canada | North America | The purpose of this study was to examine caregiver involvement in upper-limb treatment as a method to improve upper-limb function | RCT Other (questionnaire, test) | Stroke | 50 | NR | 69.2 | NR | 56 | NR | Other relation |
| Hekmatpou 2019 [25] | Iran | Asia | The study was conducted to examine the effect of patient care education on burden of care and quality of life of caregivers of stroke patients | Other (educational trial study) Questionnaire | Stroke | NR | 100 | NR | 46.8 | NR | 37 | NR |
| Kanmani 2021 [26] | India | Asia | To understand the impact of psychoeducation and psychosocial intervention on traumatic brain injury (TBI) caregivers during hospitalization | Other (descriptive study) Questionnaire | TBI | NR | 63 | NR | 39 | NR | 71.4 | Parent  Spouse or partner |
| Kim 2013 [27] | South Korea | Asia | The aims of this study were to assess the effects of such interventions among stroke patients and their primary caregivers and to evaluate the feasibility of a web-based stroke education program | Pilot study  Other (questionnaire, blood chemistry) | Stroke | 36 | 36 | 65.7 | 53.6 | 63.9 | NR | Child  Spouse or partner  Other relation |
| Kuo 2016 [28] | Taiwan | Asia | The aim of this study was to evaluate the effectiveness of a home-based oral care training programme on knowledge, attitude, self-efficacy, and practice behaviour of family caregivers | RCT Questionnaire | Stroke | 94 | 94 | NR | 53.3 | NR | 37.2 | Child  Spouse or partner  Other relation |
| Lin 2022 [29] | Australia | Australia | To evaluate the effects of a nurse-led health coaching programme for stroke survivors and family caregivers in hospital-to-home transition care | RCT Questionnaire | Stroke | 140 | 140 | 61.7 | 54.2 | 75.7 | 32.9 | Child  Spouse or partner  Other relation |
| Lindley 2017 [30] | Australia | Australia | Our primary objective was to determine whether family-led stroke rehabilitation, initiated in hospital and continued at home, would be superior to usual care in a low-resource setting | RCT Questionnaire | Stroke | 1250 | 1250 | 57.7 | NR | 67 | NR | Child  Parent  Sibling  Spouse or partner  Other family member  Other relation |
| LutzA 2022 [31] | USA | North America | To describe the effect of the COMPASS transitional care (COMPASS-TC) intervention on caregiver strain and characterize the types, duration, and intensity of caregiving | RCT Questionnaire | Stroke | 4208 | 4208 | 67.4 | NR | 49.5 | 31.9 | Child  Sibling  Spouse or partner  Other relation |
| LutzB 2022 [32] | USA | North America | The purpose of this study was to evaluate the effect of a standardized interprofessional team meeting with the caregiver also known as a “know me” meeting within 3–4 calendar days of admission compared with usual care (meeting when problems arose) on caregiver readiness for caregiving | Quasi-experimental Questionnaire | ABI | 71 | 71 | 64.6 | NR | 63.4 | NR | NR |
| Mudzi 2010 [33] | South Africa | Africa | To establish the impact of caregiver education on the morbidity of stroke survivors and on the quality of life of the stroke survivors and their caregivers | RCT Questionnaire | Stroke | 200 | 200 | 53.2 | 39.6 | 43.5 | 30 | Spouse or partner  Other relation |
| Niemeier 2019 [34] | USA | North America | The aim of the current study was to determine whether a brief, manualized intervention would meet the needs of caregivers during acute rehabilitation as well as after transition to the community | Pilot study Questionnaire | TBI | 93 | 93 | 40.1 | 51.3 | 75 | 18 | Parent  Spouse or partner  Other relation |
| Nordentoft 2022 [35] | Den-mark | Europe | The study aim is to explore patient and caregiver experiences and evaluate the relevance of and satisfaction with a multimodal rehabilitative palliative care program for patients diagnosed with an HGG and their family caregivers | Mixed methods Interviews and questionnaire | High-grade glioma | 17 | 16 | 60 (years in me-dian) | 60 (years in me-dian) | 52.9 | 31.3 | Parent  Spouse or partner  Sibling |
| Nualnetr 2010 [36] | Thailand | Asia | This study aimed to evaluate changes in motor activity, and mental and social conditions of 10 stroke survivors after participating in the 12-week community neurorehabilitation using a family-centred approach | Other (before and after study) Questionnaire | Stroke | 10 | 10 | 69.7 | 56.1 | 50 | 20 | Child  Spouse or partner |
| Osawa 2010 [37] | Japan | Asia | The present study examined whether family participation can contribute to an improvement in USN following an acute stroke | Other (intervention study) Other (questionnaire, test) | Stroke | 34 | 20 | 54.3 | NA | 47.1 | NA | NR |
| Oyesanya 2020 [38] | USA | North America | The purpose of this study was to develop and test the efficacy of a pre-discharge, medication management intervention to improve perceived knowledge and perceived confidence for post-discharge medication management for rehabilitation patients with spinal cord injuries (SCIs) and families of patients with SCIs or acquired brain injuries (ABIs) | Quasi-experimental Questionnaire | ABI | 72 | 135 | 45.5 | 50.5 | 57.4 | 24.1 | NR |
| Perlick 2013 [39] | USA | North America | This study evaluated the initial efficacy and feasibility of implementing multifamily group treatment for veterans with traumatic brain injury (TBI) | Feasibility study Other (questionnaire, test) | TBI | 14 | 12 | 36.1 | 34.4 | 78.6 | 14 | Child  Sibling  Spouse or partner |
| Philip 2018 [40] | Australia | Australia | The aim of this study was to examine the feasibility, acceptability, and preliminary effectiveness of I-CoPE for patients with high-grade glioma and their carers | Pilot study Questionnaire | High-grade glioma | 32 | 31 | 60.8 | 55.7 | 53.1 | 41.9 | Child  Parent  Spouse or partner  Other relation |
| Piil 2022 [41] | Den-mark | Europe | This study aims to understand how patients with HGG and their families experienced the course of illness and investigate the impact of family and network consultations (FNCs) on both of them | Feasibility study Interviews and questionnaire | High-grade glioma | 21 | 47 | 66 | 47 | 86 | 38 | NR |
| Pitthayapong 2017 [42] | Thailand | Asia | The purpose of this study was to evaluate the effectiveness of the post-stroke care program within the community setting in Thailand | Quasi-experimental Questionnaire | Stroke | 62 | 62 | 66.9 | 50.2 | 38.7 | 17.7 | Child  Spouse or partner  Other relation |
| Ramazanu 2021 [43] | China | Asia | Therefore, we first aimed to explore persons with stroke and their spousal caregivers' experiences of taking part in the 3Hintervention. Second, we evaluated the implementation of 3H intervention into community nursing | Qualitative Interviews | Stroke | 7 | 7 | NR | NR | 85.7 | 14.3 | Spouse or partner |
| Rasmussen 2021 [44] | Norway | Europe | To determine the effectiveness of a family-centred intervention for patients with traumatic brain injury and family members | RCT Questionnaire | TBI | 61 | 63 | 43.8 | 42.6 | 45.9 | 47.6 | Child  Parent  Spouse or partner |
| Robinson-Smith 2016 [45] | USA | North America | This mixed-methods pilot study examined the impact of a psychoeducational intervention (PEI) to expand coping skills and identify themes for focused nursing strategies to assist poststroke couples | Pilot study Other (questionnaire, interviews, observations) | Stroke | 10 | 10 | 70.4 | 70.5 | 60 | 60 | Spouse or partner |
| Shyu 2010 [46] | Taiwan | Asia | To explore the long-term effects of a discharge-preparation programme targeting Taiwanese family caregivers of older patients with stroke | Experimental study Questionnaire | Stroke | 158 | 158 | 74.2 | 47.9 | 46.2 | 39.2 | Child  Spouse or partner  Other family member |
| Stone 2014 [47] | USA | North America | The aim of this project was to test the use of web-based resources to enhance the preparedness and satisfaction of such caregivers | Other (descriptive project)  Questionnaire | Stroke and brain injury | 70 | 70 | NR | NR | 35.7 | 17.1 | Child  Parent  Spouse or partner  Other relation |
| Taricco 2014 [48] | Italy | Europe | The aim of this study was to assess whether the combination of Adapted Physical Activity (APA) and Therapeutic Patient Education (TPE) improves function and quality of life in survivors of strokes | Non-randomised controlled trial  Other (questionnaire, test) | Stroke | 229 | NR | 71 | NR | 64.2 | NR | NR |
| Tielemans 2015 [49] | The Nether-lands | Europe | To investigate the effectiveness of a self-management intervention aimed at proactive coping for stroke patients and partners, compared with an education intervention | RCT Questionnaire | Stroke | 113 | 57 | 57 | 59.2 | 52.1 | 45 | Spouse or partner |
| Vluggen 2021 [50] | The Nether-lands | Europe | The aim of this study was to evaluate the effects of this integrated programme as compared with usual care on the primary outcome daily activity level, and on the secondary outcomes functional independence, perceived quality of life and social participation of patients, and perceived care burden, objective care burden, and quality of life of their informal caregivers | RCT Questionnaire | Stroke | 190 | 172 | 78.9 | 60.8 | 39.5 | 35.5 | Sibling  Spouse or partner  Other family member  Other relation |
| Walker 2020 [51] | United Kingdom | Europe | To determine the feasibility of recruiting to and delivering a biopsychosocial intervention for carers of stroke survivors | Feasibility study Interviews and questionnaire | Stroke | 35 | 35 | 70.3 | 62.6 | 57.1 | 25.7 | Child  Parent  Sibling  Spouse or partner  Other relation |
| Wang 2015 [52] | Taiwan | Asia | This study examined whether caregiver-mediated, home-based intervention (CHI) could improve physical functioning and social participation in these patients | RCT  Other (questionnaire, test) | Stroke | 51 | 51 | 63.7 | NR | 58.8 | NR | Child  Spouse or partner  Other relation |
| Woodward 2021 [53] | USA | North America | Based on these data, the SWCM intervention was designed to address characteristics of an ideal transition by providing support, improving preparedness, identifying and ameliorating unmet needs, and providing education about stroke prevention. This manuscript reports on changes in caregiver outcomes within the first 90 days of discharge home | RCT  Questionnaire | Stroke | 169 | 169 | NR | 58.4 | NR | 23.1 | Spouse or partner  Other relation |
| Zhao 2022 [54] | China | Asia | This study aimed to develop a tailored sitting Tai Chi program for subacute stroke survivors and to examine its effects among this population | RCT Other (questionnaire, test) | Stroke | 160 | 160 | 63 | NR | 50.6 | NR | Child  Spouse or partner  Other relation |
| Zhou 2019 [55] | China | Asia | We aimed to determine the effectiveness of the program to improve the basic self-care (activities of daily living [ADL]) physical function of patients disabled after a recent stroke in a multicenter randomized controlled trial, the RECOVER (Rehabilitation Through Caregiver- Delivered Nurse-Organized Service Programs for Disabled Stroke Patients in Rural China) | Feasibility study Interviews and questionnaire | Stroke | 244 | 244 | 65.4 | 51.9 | 45.1 | 49.2 | NR |

^a^ NA: Not applicable
^b^ NR: Not reported

**Supplementary Material VII. Key characteristics of study interventions (n=46)**

| Author and year of publication | Setting | Placement of intervention in relation to course of disease | Duration of intervention | Length of follow-up (months) | Target of intervention | Involvement of relatives | Delivery mode of intervention | Providers of intervention | Type of intervention |
| --- | --- | --- | --- | --- | --- | --- | --- | --- | --- |
| Aguirrezabal 2013 [10] | Hospital | In-hospital rehabilitation | 2 hours | 6 | NR^a^ | Relatives play an active part | Person (face-to-face) Video | Nurse  PT^b^  OT^c^ | Single component |
| Araújo 2018 [11] | In-hospital and home | Rehabilitation after discharge | 3 months | 3 | Individual | Relatives play an active part | Person (face-to-face) Telephone | Nurse | Single component |
| Attard 2018 [12] | Home | The continuing developing phase | 12 weeks | 1 | Mixed | Relatives play an active part | Person (face-to-face)  Telephone | Social worker | Multicomponent |
| Avci 2021 [13] | In-hospital and home | Mixed phases | 3 months | 3 | Individual | Relatives play an active part | Person (face-to-face)  Telephone | Nurse | Multicomponent |
| Berg 2016 [14] | In-hospital and home | In-hospital rehabilitation | 8 weeks | 12 | Individual | Relatives play an active part | Person (face-to-face) Web-based or online | Nurse  PT | Single component |
| Chang 2015 [15] | Community-based | Rehabilitation after discharge | 12 weeks | None | Mixed | Relatives play an active part | Person (face-to-face) | Nurse  PT | Multicomponent |
| Chu 2020 [16] | In-hospital and home | Mixed phases | 3 days | 6 | NR | Relatives play an active part | Person (face-to-face)  Web-based or online | Nurse | Single component |
| Daviet 2022 [17] | Home | Rehabilitation after discharge | Individually according to the needs identified | None | Individual | Relatives play an active part | Person (face-to-face) | Nurse  OT  NP or P^d^  Physician  Other (physical activity teacher) | Multicomponent |
| Deyhoul 2019 [18] | In-hospital | In-hospital rehabilitation | 4 days | 2 | NR | Relatives play an active part | Person (face-to-face) | NR | Multicomponent |
| Eames 2013 [19] | In-hospital and home | In-hospital rehabilitation | 3 months | 3 | NR | Relatives play an active part | Booklet or written information  Person (face-to-face)  Telephone | OT | Multicomponent |
| Einerson 2022 [20] | Home | Mixed phases | 5 weeks | None | Individual | Intervention only for relatives | Email  Telephone  Web-based or online | OT | Multicomponent |
| Forster 2013 [21] | Other (in-hospital and residence) | In-hospital rehabilitation | NR | 12 | NR | Intervention only for relatives | NR | Other (multidisciplinary team) | Single component |
| Galvin 2014 [22] | In-hospital and home | In-hospital rehabilitation | 8 weeks | None | NR | Relatives play an active part | Person (face-to-face) | PT | Single component |
| Halkett 2023 [23] | Home | Other^e^ | 12 months | 12 | Individual | Intervention only for relatives | Person (face-to-face)  Telephone | Nurse | Multicomponent |
| Harris 2010 [24] | In-hospital and home | In-hospital rehabilitation | 4 weeks | None | Individual | Relatives play an active part | Booklet or written information  Person (face-to-face) | Other (multidisciplinary team) | Single component |
| Hekmatpou 2019 [25] | In-hospital and home | In-hospital rehabilitation | NR | None | Individual | Intervention only for relatives | Booklet or written information  Person (face-to-face)  Telephone | Nurse | Multicomponent |
| Kanmani 2021 [26] | In-hospital | Acute treatment | Individually depending on the relatives’ needs | None | Mixed | Intervention only for relatives | Person (face-to-face) | NR | Multicomponent |
| Kim 2013 [27] | Other (community-dwelling) | The continuing developing phase | 9 weeks | 3 | Individual | Relatives play an active part | Person (face-to-face)  Telephone  Web-based or online | Other (research assistant) | Multicomponent |
| Kuo 2016 [28] | Home | The continuing developing phase | 2 months | 2 | Individual | Relatives play an active part | Booklet or written information  Person (face-to-face) | Nurse | Single component |
| Lin 2022 [29] | Other (in-hospital and outpatient clinic) | In-hospital rehabilitation | 12 weeks | 6 | Individual | Relatives play an active part | Person (face-to-face)  Telephone | Nurse | Single component |
| Lindley 2017 [30] | In-hospital and home | Mixed phases | NR | 6 | NR | Relatives play an active part | Person (face-to-face)  Telephone | PT | Multicomponent |
| LutzA 2022 [31] | Other (home and clinic visits) | Mixed phases | 30 days | 3 | Individual | Relatives play an active part | Person (face-to-face)  Telephone | Nurse  Other (telephone interviewer) | Single component |
| LutzB 2022 [32] | In-hospital | In-hospital rehabilitation | NR | None | Individual | Relatives play an active part | Person (face-to-face) | Nurse  Physician  Other (case manager) | Multicomponent |
| Mudzi 2010 [33] | In-hospital | Mixed phases | Individually depending on identified needs | 12 | Individual | Relatives play an active part | Person (face-to-face) | PT  OT | Multicomponent |
| Niemeier 2019 [34] | In-hospital and home | Mixed phases | NR | 3 | NR | Intervention only for relatives | Booklet or written information Person (face-to-face) | NP or P | Multicomponent |
| Nordentoft 2022 [35] | NR | NR | 12 weeks | 3 | Mixed | Relatives play an active part | Person (face-to-face) | Nurse  PT  OT  Social worker  NP or P  Physician | Multicomponent |
| Nualnetr 2010 [36] | NR | Rehabilitation after discharge | 12 weeks | None | Individual | Relatives play an active part | Person (face-to-face) | NR | Single component |
| Osawa 2010 [37] | In-hospital | In-hospital rehabilitation | 3 weeks | None | NR | Relatives play an active part | Person (face-to-face) | NR | Single component |
| Oyesanya 2020 [38] | In-hospital | In-hospital rehabilitation | 1 hour | 2 | NR | Relatives play an active part | Booklet or written information Person (face-to-face)  Web-based or online | NR | Single component |
| Perlick 2013 [39] | NR | The continuing developing phase | 9 months | None | Mixed | Intervention only for relatives | Person (face-to-face) | Other (multifamily group clinicians and supervisors, group leaders) | Multicomponent |
| Philip 2018 [40] | Other (in-hospital and radiotherapy) | Other^f^ | NR | 3 | Individual | Relatives play an active part | Person (face-to-face)  Telephone | Nurse  Other (care coordinator) | Multicomponent |
| Piil 2022 [41] | NR | NR | 1 year | NR | NR | Relatives play an active part | NR | Nurse | NR |
| Pitthayapong 2017 [42] | Home | The continuing developing phase | 4 weeks | 2 | Individual | Relatives play an active part | Person (face-to-face) | Other (health care provider) | Multicomponent |
| Ramazanu 2021 [43] | In-hospital | Rehabilitation after discharge | 3 weeks | None | Mixed | Relatives play an active part | Person (face-to-face) | Nurse | Multicomponent |
| Rasmussen 2021 [44] | Other (in-hospital and home or appropriate municipal premises) | Rehabilitation after discharge | 8 weeks | 8 | Group | Relatives play an active part | Person (face-to-face) | Nurse  PT  OT | Multicomponent |
| Robinson-Smith 2016 [45] | Outpatient clinic | In-hospital rehabilitation | NR | None | Individual | Relatives play an active part | Person (face-to-face) | Nurse | Multicomponent |
| Shyu 2010 [46] | In-hospital and home | In-hospital rehabilitation | Individually depending on duration of admission | 12 | Individual | Relatives play an active part | Person (face-to-face)  Telephone | Nurse | Multicomponent |
| Stone 2014 [47] | In-hospital | In-hospital rehabilitation | NR | None | NR | Intervention only for relatives | Person (face-to-face)  Web-based or online | Nurse | Multicomponent |
| Taricco 2014 [48] | Home and community | Rehabilitation after discharge | NR | 12 | Mixed | Relatives play an active part | Booklet or written information Person (face-to-face) | PT  Physician | Multicomponent |
| Tielemans 2015 [49] | Outpatient clinic | Rehabilitation after discharge | 12 weeks | 9 | Group | Relatives play an active part | Booklet or written information Person (face-to-face) | OT  NP or P | Multicomponent |
| Vluggen 2021 [50] | Home and community | Mixed phases | Individually depending on the care needs of the patient | 12 | Individual | Relatives play an active part | Person (face-to-face) | PT  OT  NP or P  Speech-language therapist  Physician  Other (stroke coordinator) | Multicomponent |
| Walker 2020 [51] | Community-based | Rehabilitation after discharge | 6 weeks | 6 | Mixed | Intervention only for relatives | Booklet or written information Person (face-to-face) | NP or P | Multicomponent |
| Wang 2015 [52] | Home | Rehabilitation after discharge | 12 weeks | 3 | Individual | Relatives play an active part | Person (face-to-face) | PT | Single component |
| Woodward 2021 [53] | Home | Mixed phases | 60 days | 3 | Individual | Relatives play an active part | Booklet or written information Person (face-to-face)  Telephone | NR | Multicomponent |
| Zhao 2022 [54] | Other (In-hospital and home or rehabilitation) | Mixed phases | 12 weeks | 4 | NR | Relatives play an active part | Person (face-to-face) | Nurse  Other (Tai Chi master) | Single component |
| Zhou 2019 [55] | In-hospital and home | Mixed phases | 3 days | 6 | Individual | Relatives play an active part | Person (face-to-face) | Nurse | Multicomponent |

^a^ NR: Not reported ^b^ PT: Physiotherapist
^c^ OT: Occupational therapist
^d^ NP or P: Neuropsychologist or psychologist
^e^ Other: Undergoing active treatment
^f^ Other: 3 transitions in illness course: at diagnosis, following the diagnostic hospitalization, following radiotherapy

**Supplementary Material VIII. Components in study interventions**

**Components**

|  | **Number of components** | **Coaching** | **Cognitive-behavioral** | **Communication** | **Coordination of care** | **Coping** | **Education** | **Emotional or cognitive** | **Goal setting** | **Information** | **Management or self-management** | **Physical activity or training** | **Problem-solving** | **Psychological** | **Shared decision making** | **Skills training** | **Social** | **Support** |
| --- | --- | --- | --- | --- | --- | --- | --- | --- | --- | --- | --- | --- | --- | --- | --- | --- | --- | --- |
| Aguirrezabal 2013 [10] | 1 |  |  |  |  |  | X |  |  |  |  |  |  |  |  |  |  |  |
| Araújo 2018 [11] | 1 |  |  |  |  |  | X |  |  |  |  |  |  |  |  |  |  |  |
| Attard 2018 [12] | 6 |  |  | X |  |  | X |  |  |  |  | X |  | X |  |  | X | X |
| Avci 2021 [13] | 2 |  |  |  |  |  | X |  |  |  |  | X |  |  |  |  |  |  |
| Berg 2016 [14] | 1 |  |  |  |  |  |  |  |  |  |  | X |  |  |  |  |  |  |
| Chang 2015 [15] | 3 |  |  |  |  |  | X |  |  | X |  | X |  |  |  |  |  |  |
| Chu 2020 [16] | 1 |  |  |  |  |  |  |  |  |  |  | X |  |  |  |  |  |  |
| Daviet 2022 [17] | 6 |  |  | X |  |  | X | X |  | X |  | X |  |  |  |  | X |  |
| Deyhoul 2019 [18] | 2 |  |  |  |  |  | X |  |  | X |  |  |  |  |  |  |  |  |
| Eames 2013 [19] | 2 |  |  |  |  |  | X |  |  |  |  |  |  |  |  |  |  | X |
| Einerson 2022 [20] | 3 |  |  |  |  |  | X |  | X |  |  |  |  |  |  | X |  |  |
| Forster 2013 [21] | 1 |  |  |  |  |  |  |  |  |  |  | X |  |  |  |  |  |  |
| Galvin 2014 [22] | 1 |  |  |  |  |  |  |  |  |  |  | X |  |  |  |  |  |  |
| Halkett 2023 [23] | 5 |  |  | X |  |  | X | X |  | X |  |  |  |  |  |  |  | X |
| Harris 2010 [24] | 1 |  |  |  |  |  |  |  |  |  |  | X |  |  |  |  |  |  |
| Hekmatpou 2019 [25] | 2 |  |  |  |  |  | X |  |  |  |  | X |  |  |  |  |  |  |
| Kanmani 2021 [26] | 3 |  |  |  |  |  | X |  |  | X |  |  |  | X |  |  |  |  |
| Kim 2013 [27] | 2 |  |  |  |  |  | X |  |  |  |  |  |  |  |  |  |  | X |
| Kuo 2016 [28] | 1 |  |  |  |  |  | X |  |  |  |  |  |  |  |  |  |  |  |
| Lin 2022 [29] | 1 | X |  |  |  |  |  |  |  |  |  |  |  |  |  |  |  |  |
| Lindley 2017 [30] | 2 |  |  |  |  |  | X |  |  |  |  | X |  |  |  |  |  |  |
| LutzA 2022 [31] | 1 |  |  |  |  |  |  |  |  |  |  |  |  |  |  |  |  | X |
| LutzB 2022 [32] | 2 |  |  |  |  |  | X |  |  | X |  |  |  |  |  |  |  |  |
| Mudzi 2010 [33] | 4 |  |  |  |  |  | X |  |  | X |  | X |  |  |  | X |  |  |
| Niemeier 2019 [34] | 3 |  |  |  |  |  | X | X |  | X |  |  |  |  |  |  |  |  |
| Nordentoft 2022 [35] | 4 |  |  |  |  | X | X |  |  |  |  | X |  |  |  |  |  | X |
| Nualnetr 2010 [36] | 1 |  |  |  |  |  |  |  |  |  |  | X |  |  |  |  |  |  |
| Osawa 2010 [37] | 1 |  |  |  |  |  |  |  |  |  |  | X |  |  |  |  |  |  |
| Oyesanya 2020 [38] | 1 |  |  |  |  |  | X |  |  |  |  |  |  |  |  |  |  |  |
| Perlick 2013 [39] | 3 |  |  |  |  |  | X |  | X |  |  |  | X |  |  |  |  |  |
| Philip 2018 [40] | 3 |  |  |  | X |  |  | X |  | X |  |  |  |  |  |  |  |  |
| Piil 2022 [41] | NR^a^ |  |  |  |  |  |  |  |  |  |  |  |  |  |  |  |  |  |
| Pitthayapong 2017 [42] | 4 |  |  |  |  |  | X | X |  | X |  | X |  |  |  |  |  |  |
| Ramazanu 2021 [43] | 3 |  |  |  |  |  |  |  |  |  |  |  |  |  | X | X |  | X |
| Rasmussen 2021 [44] | 4 |  | X | X |  |  |  | X |  |  |  |  |  |  |  | X |  |  |
| Robinson-Smith 2016 [45] | 5 |  |  | X |  | X | X | X |  |  |  |  |  |  |  |  |  | X |
| Shyu 2010 [46] | 2 |  |  |  |  |  | X |  |  | X |  |  |  |  |  |  |  |  |
| Stone 2014 [47] | 2 |  |  | X |  |  |  |  |  |  | X |  |  |  |  |  |  |  |
| Taricco 2014 [48] | 2 |  |  |  |  |  | X |  |  |  |  | X |  |  |  |  |  |  |
| Tielemans 2015 [49] | 4 |  |  |  |  |  | X |  |  |  | X | X |  |  |  |  |  | X |
| Vluggen 2021 [50] | 5 |  | X |  |  |  | X | X |  |  |  | X |  | X |  |  |  |  |
| Walker 2020 [51] | 5 |  |  |  |  | X | X | X |  | X |  | X |  |  |  |  |  |  |
| Wang 2015 [52] | 1 |  |  |  |  |  |  |  |  |  |  | X |  |  |  |  |  |  |
| Woodward 2021 [53] | 4 |  |  |  | X |  | X |  |  | X | X |  |  |  |  |  |  |  |
| Zhao 2022 [54] | 1 |  |  |  |  |  |  |  |  |  |  | X |  |  |  |  |  |  |
| Zhou 2019 [55] | 3 |  |  |  |  |  | X |  |  | X |  | X |  |  |  |  |  |  |

^a^ NR: Not reported

**Supplementary Material IX. Outcomes and outcome measurements in included studies (n=46)**

| **Author and year of publication** | **Outcomes related to relatives** | **Outcomes** | **Outcome measurements** |
| --- | --- | --- | --- |
| Aguirrezabal 2013 [10] | *No outcomes related to relatives* |  | |
| Araújo 2018 [11] | Yes (P) | Skills (P^a^)  Caregiver burden (S^b^)  Health condition | ECPICID-AVC^c^ (P)  QASCI (S)  SF-36 |
| Attard 2018 [12] | Yes | Skills  Caregiver burden  Psychological health | MSC  BCOS  GHQ-12 |
| Avci 2021 [13] | *Qualitative study* |  | |
| Berg 2016 [14] | Yes | Caregiver burden (S) | HADS (S), GSES (S), FSS (S), CarerQoL (S), eCSI (S) |
| Chang 2015 [15] | Yes | Satisfaction, taking care Satisfaction, care service | CSS-KV MCSS |
| Chu 2020 | Yes | Caregiver burden (S) | CBI (S) |
| Daviet 2022 | Yes | Caregiver burden (S) Quality of life (S) | mZBI (S) EQ5D (S) |
| Deyhoul 2019 | Yes | Caregiver burden | CBI |
| Eames 2013 | Yes (P) | Knowledge (P) Self-efficacy (S) Anxiety and depression (S) Caregiver burden  Information | KoSQ-25 (P) QUEST-9 (S) HADS (S) CSI INFO-QUEST |
| Einerson 2022 | *No outcomes related to relatives* | | |
| Forster 2013 | Yes (P) | Caregiver burden (P) Anxiety and depression (S) Quality of life (S) Life years (S) Resource use (S)  Mortality (S) | CBS (P)  HADS (S) EQ5D (S) QALY (S) CSRI (S) Death (S) |
| Galvin 2014 | *Qualitative study* |  | |
| Halkett 2023 | Yes (P) | Preparedness for caregiving (P) Distress (P) Anxiety and depression (S) Quality of life (S) Carer competence (S)  Caregiver strain (S) | PCS (P)  DT (P) HADS (S) CQoLI-C (S) CCS (S) mCSI (S) |
| Harris 2010 | *No outcomes related to relatives* | | |
| Hekmatpou 2019 | Yes | Caregiver burden  Quality of life | ZBI  SF-36 |
| Kanmani 2021 | *No outcomes related to relatives* | | |
| Kim 2013 | Yes (P) | Stable self-image (P) | CGMS (P) |
| Kuo 2016 | Yes | Knowledge  Attitude  Self-efficacy  Behavior | KoOC-44 ATOC-19  SEQ-34  BoOC-26 |
| Lin 2022 | Yes | Caregiver burden (S) | mCSI (S) |
| Lindley 2017 | Yes | Anxiety and depression (S) | CBS (S), HADS (S) |
| LutzA 2022 | Yes | Caregiver burden Assistance with ADL | mCSI  QCaADL |
| LutzB 2022 | Yes | Preparedness for caregiving | PCS |
| Mudzi 2010 | Yes | Quality of life  Caregiver burden | EQ5D  CSI |
| Niemeier 2019 | Yes | Needs  Caregiver burden  Knowledge  Distress | FNQR  ZBI  BICKA  BSI-18 |
| Nordentoft 2022 | *No outcomes related to relatives* | | |
| Nualnetr 2010 | *No outcomes related to relatives* | | |
| Osawa 2010 | *No outcomes related to relatives* | | |
| Oyesanya 2020 | Yes (P) | Management, medicine (P) | Survey-10 (P) |
| Perlick 2013 | Yes | Caregiver burden  Coping efficacy  Anger management  Network and support | CBI  FES  AXS-24  ADSSI |
| Philip 2018 | Yes | Needs  Quality of life  Preparedness for caregiving | CSNAT, PINQ  CQoLI-C  PCS |
| Piil 2022 | Yes | Caregiver burden | CBI |
| Pitthayapong 2017 | Yes | Skills | PCSC |
| Ramazanu 2021 | *Qualitative study* | | |
| Rasmussen 2021 | Yes (P) | Quality of life (P) Caregiver burden (P) Cohesion and flexibility (S) | SF-36 (P)  CBS (P)  FACES (S) |
| Robinson-Smith 2016 | Yes | Communication | DCI |
| Shyu 2010 | Yes | Quality of life | SF-36 |
| Stone 2014 | Yes (P) | Preparedness for caregiving (P) | PCS (P) |
| Taricco 2014 | Yes | Caregiver burden 2 | CSI 2 |
| Tielemans 2015 | Yes (P) | Coping competence (P) Self-efficacy (S) Emotional functioning (S)  Caregiver burden (S) | UPCCS (P)  GSES-DV (S) HADS (S) CSI (S) |
| Vluggen 2021 | Yes | Caregiver burden (S) Quality of life (S)  Care load (S) | SB-VAS (S) CarerQoL (S) E-iBMG (S) |
| Walker 2020 | Yes | Anxiety and depression  Quality of life  Caregiver burden | HADS  EQ5D  CBS |
| Wang 2015 | Yes | Caregiver burden | CBS |
| Woodward 2021 | Yes (P) | Caregiver role (P) Depressive symptoms (P) Support (S) | BCOS (P)  PHQ-9 (P)  PROMIS-E (S), PROMIS-I (S) |
| Zhao 2022 | *No outcomes related to relatives* | | |
| Zhou 2019 | Yes | Caregiver burden | CBIndex (S) |

^a^ ’P’ indicates that the outcome is a primary outcome
^b^ ‘S’ indicates that the outcome is a secondary outcome
^c^ See Appendix IX for scales abbreviations

**Supplementary Material X**

**Description of outcome measurement in case of one outcome and one associated outcome measurement**

Assistance with ADL: QCaADL
Emotional functioning: HADS
Health condition: SF-36
Psychological health: GHQ-12
Caregiver strain: mCSI
Care load: E-iBMG
Caregiver role: BCOS
Satisfaction, taking care: CSS-KV
Satisfaction, care service: MCSS

Depressive symptoms: PHQ-9
Life years: QALY
Resource use: CSRI
Mortality: Death
Attitude: ATOC-19
Behavior: BoOC-26
Carer competence: CCS
Stable self-image: CGMS
Information: INFO-QUEST


Management, medicine: Survey-10
Coping efficacy: FES
Anger management: AXS-24
Network and support: ADSSI
Cohesion and flexibility: FACES
Communication: DCI
Coping competence: UPCCS

**Abbreviations**

| ADSSI: The Abbreviated Duke Social Support Index |
| --- |
| ATOC-19: 19-item Attitude towards Oral Care Questionnaire |
| AXS-24: The 24-item AX Scale |
| BCOS: Bakas Caregiving Outcomes Scale |
| BICKA: Brain Injury Caregiver Knowledge Assessment (developed for the intervention) |
| BoOC-26: 26-item Behavior of Oral Care Questionnaire |
| BSI-18: Brief Symptom Inventory-18 |
| CarerQoL: Carer Quality of Life Scale |
| CBI: Caregiver Burden Inventory |
| CBIndex: Caregiver Burden Index |
| CBS: Caregiver Burden scale |
| CCS: Carer Competence Scale |
| CGMS: Care Giving Mastery Scale |
| CSI: Caregiver Strain Index |
| CQoLI-C: Caregiver Quality of Life Index-Cancer |
| CSNAT: Carer Support Care Needs Assessment Tool |
| CSRI: Self-complete Client Service Receipt Inventory |
| CSS-KV: Caregiver Satisfaction scale - Korean Version |
| DCI: Dyadic Coping Instrument |
| DT: Distress Thermometer |
| ECPICID-AVC: Skills Scale of Informal Caregivers of Dependent Older People Post-stroke |
| eCSI: expanded Caregiver Strain Index |
| E-iBMG: Erasmus iBMG |
| EQ5D: European Quality of Life, 5-Dimensions |
| FACES: The Family Adaptability and Cohesion Evaluation Scale |
| FES: Family Empowerment Scale |
| FNQR: Family Needs Questionnaire-Revised |
| FSS: Fatigue Severity Scale |
| GCQ-12: General Health Questionnaire-12 |
| GSES: General Self-efficacy Scale |
| GSES-DV: General Self-efficacy Scale - Dutch Version |
| HADS: Hospital Anxiety and Depression Scale |
| INFO-QUEST: 10-point Likert Scale regarding information (being informed, importance of information and satisfaction with information received) |
| KoOC-44: 44-item Knowledge of Oral Care Questionnaire |
| KoSQ-25: 25-item Knowledge of Stroke Questionnaire |
| mCSI: modified Carer Strain Index |
| MCSS: The Modified Caregiver Satisfaction Scale |
| MSC: Measure of skill in Supported Conversation |
| mZBI: MiniZarit Burden Interview, 7-item |
| PCS: Preparedness for Caregiving Scale |
| PCSC: Post-stroke Care Skills Checklist (developed for the intervention) |
| PHQ-9: Patient Health Questionnaire-9 |
| PINQ: Patient Information Needs Questionnaire |
| PROMIS-E: PROMIS Emotional |
| PROMIS-I: PROMIS Informational |
| QALY: Quality-adjusted life-years |
| QASCI: Evaluation Questionnaire on Informal Caregiver's Burden |
| QCaADL: Questionnaire on caregiver assistance with Activities of Daily Living (ADL) and instrumental ADL (developed for the intervention) |
| QUEST-9: 9-item questionnaire on self-efficacy (developed for the intervention) |
| SB-VAS: Self-rated Burden on a 10-point Visuel Analog Scale |
| SEQ-34: 34-item questionnaire on self-efficacy of oral care |
| SF-36: 36-item Short Form Health Survey |
| Survey-10: 10-item survey assessing caregivers’ knowledge and perceived confidence for post-discharge medication management (developed for the intervention) |
| UPCCS: Utrecht Proactive Coping Competence Scale |
| ZBI: Zarit Burden Interview |

**References**

1. Bramer, W.M., et al., *Optimal database combinations for literature searches in systematic reviews: a prospective exploratory study.* Syst Rev, 2017. **6**(1): p. 245.

2. Blanton, S., et al., *A web-based carepartner-integrated rehabilitation program for persons with stroke: study protocol for a pilot randomized controlled trial.* Pilot Feasibility Stud, 2019. **5**: p. 58.

3. Brasier, C., et al., *The Stroke and Carer Optimal Health Program (SCOHP) to enhance psychosocial health: study protocol for a randomized controlled trial.* Trials, 2016. **17**(1): p. 446.

4. Elsheikh, M.A., et al., *Effect of a tailored multidimensional intervention on the care burden among family caregivers of stroke survivors: study protocol for a randomised controlled trial.* BMJ Open, 2020. **10**(12): p. e041637.

5. Mulder, M., et al., *Can telerehabilitation services combined with caregiver-mediated exercises improve early supported discharge services poststroke? A study protocol for a multicentre, observer-blinded, randomized controlled trial.* BMC Neurol, 2022. **22**(1): p. 29.

6. Ownsworth, T., et al., *Evaluation of a telehealth psychological support intervention for people with primary brain tumour and their family members: Study protocol for a randomised controlled trial.* Eur J Cancer Care (Engl), 2019. **28**(4): p. e13132.

7. Vloothuis, J., et al., *Caregiver-mediated exercises with e-health support for early supported discharge after stroke (CARE4STROKE): study protocol for a randomized controlled trial.* BMC Neurol, 2015. **15**: p. 193.

8. Worrall, L., et al., *Reducing the psychosocial impact of aphasia on mood and quality of life in people with aphasia and the impact of caregiving in family members through the Aphasia Action Success Knowledge (Aphasia ASK) program: study protocol for a randomized controlled trial.* Trials, 2016. **17**: p. 153.

9. Yan, L.L., et al., *A randomized controlled trial on rehabilitation through caregiver-delivered nurse-organized service programs for disabled stroke patients in rural china (the RECOVER trial): design and rationale.* Int J Stroke, 2016. **11**(7): p. 823-30.

10. Aguirrezabal, A., et al., *Effects of information and training provision in satisfaction of patients and carers in stroke rehabilitation.* NeuroRehabilitation, 2013. **33**(4): p. 639-47.

11. Araújo, O., et al., *Training informal caregivers to care for older people after stroke: A quasi-experimental study.* J Adv Nurs, 2018.

12. Attard, M.C., et al., *The efficacy of an inter-disciplinary community aphasia group for living well with aphasia.* Aphasiology, 2018. **32**(2): p. 105-138.

13. Avci, Y.D. and S. Gozum, *Assessment of the Transitional Care Model-Based TEMpEST Program by Caregivers of Stroke Patients: A Qualitative Study.* Home Healthc Now, 2021. **39**(4): p. 215-219.

14. van den Berg, M., et al., *Early Supported Discharge by Caregiver-Mediated Exercises and e-Health Support After Stroke: A Proof-of-Concept Trial.* Stroke, 2016. **47**(7): p. 1885-92.

15. Chang, A.K., et al., *A family involvement and patient-tailored health management program in elderly Korean stroke patients' day care centers.* Rehabil Nurs, 2015. **40**(3): p. 179-87.

16. Chu, K., et al., *Feasibility of a Nurse-Trained, Family Member-Delivered Rehabilitation Model for Disabled Stroke Patients in Rural Chongqing, China.* J Stroke Cerebrovasc Dis, 2020. **29**(12): p. 105382.

17. Daviet, J.C., et al., *Individualized home-based rehabilitation after stroke in France: a pragmatic study of a community stroke rehabilitation team.* Can J Neurol Sci, 2022: p. 1-6.

18. Deyhoul, N., et al., *The effect of family-centered empowerment program on the family caregiver burden and the activities of daily living of Iranian patients with stroke: a randomized controlled trial study.* Aging Clin Exp Res, 2020. **32**(7): p. 1343-1352.

19. Eames, S., et al., *Randomised controlled trial of an education and support package for stroke patients and their carers.* BMJ Open, 2013. **3**(5).

20. Einerson, J., et al., *Piloting GETCare: A Goal-Based Education and Skills Training Program for Caregivers.* Rehabil Nurs, 2022. **47**(6): p. 220-227.

21. Forster, A., et al., *A structured training programme for caregivers of inpatients after stroke (TRACS): a cluster randomised controlled trial and cost-effectiveness analysis.* Lancet, 2013. **382**(9910): p. 2069-76.

22. Galvin, R., E. Stokes, and T. Cusack, *Family-Mediated Exercises (FAME): an exploration of participant's involvement in a novel form of exercise delivery after stroke.* Top Stroke Rehabil, 2014. **21**(1): p. 63-74.

23. Halkett, G.K.B., et al., *Carer preparedness improved by providing a supportive educational intervention for carers of patients with high-grade glioma: RCT results.* J Neurooncol, 2023. **161**(3): p. 501-513.

24. Harris, J.E., et al., *The role of caregiver involvement in upper-limb treatment in individuals with subacute stroke.* Phys Ther, 2010. **90**(9): p. 1302-10.

25. Hekmatpou, D., E. Mohammad Baghban, and L. Mardanian Dehkordi, *The effect of patient care education on burden of care and the quality of life of caregivers of stroke patients.* J Multidiscip Healthc, 2019. **12**: p. 211-217.

26. Kanmani, T.R., et al., *Tailor-Made Psychosocial Intervention for the Caregivers in Emergency and Trauma Care Center.* J Neurosci Rural Pract, 2021. **12**(2): p. 290-294.

27. Kim, J.I., S. Lee, and J.H. Kim, *Effects of a web-based stroke education program on recurrence prevention behaviors among stroke patients: a pilot study.* Health Educ Res, 2013. **28**(3): p. 488-501.

28. Kuo, Y.W., et al., *A home-based training programme improves family caregivers' oral care practices with stroke survivors: a randomized controlled trial.* Int J Dent Hyg, 2016. **14**(2): p. 82-91.

29. Lin, S., et al., *Nurse-led health coaching programme to improve hospital-to-home transitional care for stroke survivors: A randomised controlled trial.* Patient Educ Couns, 2022. **105**(4): p. 917-925.

30. *Family-led rehabilitation after stroke in India (ATTEND): a randomised controlled trial.* Lancet, 2017. **390**(10094): p. 588-599.

31. Lutz, B.J., et al., *Familial caregiving following stroke: findings from the comprehensive post-acute stroke services (COMPASS) pragmatic cluster-randomized transitional care study.* Top Stroke Rehabil, 2023. **30**(5): p. 436-447.

32. Lutz, A.M., et al., *Implementation of Interprofessional Meetings Preparing Caregivers of Patients With Brain Injury for Discharge: A Pilot Study.* Prof Case Manag, 2022. **27**(5): p. 239-245.

33. Mudzi, W., *Impact of caregiver education on stroke survivors and their caregivers*, in *Faculty of Health Sciences*. 2010, University of the Witwatersrand: Johannesburg. p. 357.

34. Niemeier, J.P., et al., *A Randomized Controlled Pilot Study of a Manualized Intervention for Caregivers of Patients With Traumatic Brain Injury in Inpatient Rehabilitation.* Arch Phys Med Rehabil, 2019. **100**(4s): p. S65-s75.

35. Nordentoft, S., et al., *Evaluation of a multimodal rehabilitative palliative care programme for patients with high-grade glioma and their family caregivers.* Scand J Caring Sci, 2022. **36**(3): p. 815-829.

36. Nualnetr, N., W. Srisoparb, and W. Eungpinichpong, *The application of community neurorehabilitation using a family-centered approach to persons with disability: a case study in stroke survivors.* Asia Pacific Disability Rehabilitation Journal, 2010. **21**(1): p. 71-79.

37. Osawa, A. and S. Maeshima, *Family participation can improve unilateral spatial neglect in patients with acute right hemispheric stroke.* Eur Neurol, 2010. **63**(3): p. 170-5.

38. Oyesanya, T.O., et al., *A Medication Management Intervention for Patients and Families with SCI and ABI.* West J Nurs Res, 2020. **42**(12): p. 1113-1128.

39. Perlick, D.A., et al., *Implementation of multifamily group treatment for veterans with traumatic brain injury.* Psychiatr Serv, 2013. **64**(6): p. 534-40.

40. Philip, J., et al., *I-CoPE: A pilot study of structured supportive care delivery to people with newly diagnosed high-grade glioma and their carers.* Neurooncol Pract, 2019. **6**(1): p. 61-70.

41. Piil, K., et al., *FAMILY-CENTRED CARE IN NEURO-ONCOLOGY: A LONGITUDINAL MIXED-METHODS FEASIBILITY STUDY.* Neuro-Oncology, 2022. **24**(Supplement 2): p. ii45.

42. Pitthayapong, S., et al., *A Community Based Program for Family Caregivers for Post Stroke Survivors in Thailand.* Asian Nurs Res (Korean Soc Nurs Sci), 2017. **11**(2): p. 150-157.

43. Ramazanu, S., V.C. Lim Chiang, and M. Valimaki, *The Experiences and Evaluation of a Complex Intervention for Couples Coping With Stroke.* J Neurosci Nurs, 2021. **53**(1): p. 18-23.

44. Rasmussen, M.S., et al., *The effectiveness of a family-centred intervention after traumatic brain injury: A pragmatic randomised controlled trial.* Clin Rehabil, 2021. **35**(10): p. 1428-1441.

45. Robinson-Smith, G., et al., *Couples' Coping After Stroke-A Pilot Intervention Study.* Rehabil Nurs, 2016. **41**(4): p. 218-29.

46. Shyu, Y.I., et al., *A clinical trial of an individualised intervention programme for family caregivers of older stroke victims in Taiwan.* J Clin Nurs, 2010. **19**(11-12): p. 1675-85.

47. Stone, K., *Enhancing preparedness and satisfaction of caregivers of patients discharged from an inpatient rehabilitation facility using an interactive website.* Rehabil Nurs, 2014. **39**(2): p. 76-85.

48. Taricco, M., et al., *Impact of adapted physical activity and therapeutic patient education on functioning and quality of life in patients with postacute strokes.* Neurorehabil Neural Repair, 2014. **28**(8): p. 719-28.

49. Tielemans, N.S., et al., *Effectiveness of the Restore4Stroke self-management intervention "Plan ahead!": A randomized controlled trial in stroke patients and partners.* J Rehabil Med, 2015. **47**(10): p. 901-9.

50. Vluggen, T., et al., *Effectiveness of an integrated multidisciplinary geriatric rehabilitation programme for older persons with stroke: a multicentre randomised controlled trial.* BMC Geriatr, 2021. **21**(1): p. 134.

51. Walker, M.F., et al., *Biopsychosocial intervention for stroke carers (BISC): results of a feasibility randomised controlled trial and nested qualitative interview study.* Clin Rehabil, 2020. **34**(10): p. 1268-1281.

52. Wang, T.C., et al., *Caregiver-mediated intervention can improve physical functional recovery of patients with chronic stroke: a randomized controlled trial.* Neurorehabil Neural Repair, 2015. **29**(1): p. 3-12.

53. Woodward, A.T., et al., *Effect of transitional care stroke case management interventions on caregiver outcomes: the MISTT randomized trial.* Soc Work Health Care, 2021: p. 1-14.

54. Zhao, J., et al., *Tailored Sitting Tai Chi Program for Subacute Stroke Survivors: A Randomized Controlled Trial.* Stroke, 2022. **53**(7): p. 2192-2203.

55. Zhou, B., et al., *Caregiver-Delivered Stroke Rehabilitation in Rural China.* Stroke, 2019. **50**(7): p. 1825-1830.
